# Teaching Intersectionality of Sexual Orientation, Gender Identity, and Race/Ethnicity in a Health Disparities Course

**DOI:** 10.15766/mep_2374-8265.10970

**Published:** 2020-07-31

**Authors:** Stephanie Bi, Monica B. Vela, Aviva G. Nathan, Kathryn E. Gunter, Scott C. Cook, Fanny Y. López, Robert S. Nocon, Marshall H. Chin

**Affiliations:** 1 Student, University of Chicago Pritzker School of Medicine; 2 Professor, Department of Medicine, University of Chicago; Associate Dean for Multicultural Affairs, University of Chicago Pritzker School of Medicine; 3 Senior Research Project Manager, Department of Medicine, University of Chicago; 4 Deputy Director, Department of Medicine, University of Chicago; 5 Clinical Psychologist, Department of Medicine, University of Chicago; 6 Project Manager, Office of Diversity, Equity and Inclusion, Dominican University; Adjunct Instructor, Office of Diversity, Equity and Inclusion, Dominican University; 7 Senior Health Services Researcher, Department of Medicine, University of Chicago; 8 Richard Parrillo Family Professor of Healthcare Ethics, Department of Medicine, University of Chicago

**Keywords:** LGBTQ, Sexual and Gender Minorities, Human Sexuality, Intersectionality, Race, Ethnicity, Health Disparities, Identity, Gender Identity, Cultural Competence, Diversity, Inclusion, Health Equity, Anti-racism

## Abstract

**Introduction:**

Intersectionality considers how different identities simultaneously affect an individual's experiences. Those of multiple minority statuses may experience effects of intersecting systems of oppression. Most health disparities curricula do not focus on intersectionality. We studied the impact of an innovative module teaching intersectionality of sexual orientation, gender identity, and race/ethnicity issues in the required Pritzker School of Medicine course Health Care Disparities: Equity and Advocacy.

**Methods:**

A short lecture reviewed sexual and gender minority (SGM) health disparities, intersectionality, minority stress, and shared decision making (SDM) to establish shared language among 83 first-year medical students. Students then viewed four videos of SGM patients of color (POC) describing their health care experiences, each followed by moderated discussion about how compounded minority stress affects lived experiences and health and how to improve SDM for SGM POC. One video interviewee attended the session and answered students’ questions. Evaluation was performed using pre- and postsurveys.

**Results:**

Feeling somewhat/completely confident in defining intersectionality increased from 57% to 96%. Prior to the session, 62% of respondents reported feeling somewhat/completely confident in identifying barriers to care for SGM patients, and 92% after. Thirty-three percent felt somewhat/completely confident in asking SGM patients about their identities before the session, and 81% after. Eighty-four percent rated the session as very good or excellent.

**Discussion:**

The session was well received, improved student knowledge of intersectionality, and improved confidence in communicating with and caring for SGM patients. Future iterations could include condensing the lecture and including a patient panel and/or small-group discussion.

## Educational Objectives

By the end of this session, learners will be able to:

1. Define intersectionality, minority stress, and shared decision making.
2. Identify barriers to high-quality care for sexual and gender minority (SGM) patients of color.
3. Reflect on their own biases surrounding SGM patients of color.
4. Feel more confident exploring the lived experiences of SGM patients of color, including health care.

## Introduction

Sexual and gender minorities (SGM) face unique health care challenges and experience considerable health disparities.^1,2^ The AAMC's 2014 *Implementing Curricular and Institutional Climate Changes to Improve Health Care for Individuals Who Are LGBT, Gender Nonconforming, or Born With DSD* report encourages inclusion of SGM health curricula in medical schools.^3^ While this recommendation is an important step, the mean of national medical preclinical curricula devoted to SGM health was only 5 hours in 2011.^4,5^ In addition, when we reviewed literature in *MedEdPORTAL* and PubMed, we found that the topic of intersectionality was often absent from SGM health curricula in medical schools. Intersectionality considers multiple identities at once, rather than in isolation. Multiple systems of oppression perpetuate discrimination and disadvantage based on factors such as race, class, sex, sexual orientation, and gender identity.^6^ Barriers to inclusion of curricula about SGM racial and ethnic minorities include clinicians’ limited knowledge of and access to SGM racial and ethnic minority communities.^7^ SGM racial and ethnic minorities are a population that experiences both racism in SGM communities and homophobia/transphobia in racial/ethnic minority communities. These persons have had little input in the development of medical education. Discussion of SGM and racial/ethnic minority issues can be emotionally charged for both educators and learners and therefore must be carefully navigated. Our interactive multimedia session seeks to address this gap in medical education.

The University of Chicago Pritzker School of Medicine's required 8-week course for first-year medical students, Health Care Disparities: Equity and Advocacy, directed by coauthor Monica B. Vela, educates students about the existence and magnitude of health care disparities as well as the physician's role in advocacy in the United States for patient populations at risk for these disparities. Modules address multiple demographic groups, including Native Americans, nonnative English speakers, and African Americans, and issues such as physician bias, institutionalized racism, and social determinants of health.^8–10^ Our team reshaped the SGM module of the course. Based upon the initial success of the session, we also taught the module in shortened forms to physicians, residents, and medical center staff at the Department of Medicine grand rounds, and to accepted medical school applicants revisiting the Pritzker School of Medicine in an interactive grand rounds–style auditorium setting.

To our knowledge, only one other published educational innovation has taught SGM health care disparities with a focus on intersectionality. Potter, Burnett-Bowie, and Potter created a 2-hour module featuring a didactic presentation, role-play scenarios, and small-group work.^11^ That module increased reported awareness of the health impacts of identity and intersectionality. Our 2.5-hour module with video interviews of SGM patients of color (POC) could complement modules that teach general SGM or race/ethnicity issues or Potter and colleagues’ module. The videos showcase diverse voices and topics and eliminate logistical challenges in recruiting patients for a teaching session. Our overall session employs lecture, patient videos, discussion of the videos, and a live patient storytelling and question-and-answer component.

## Methods

### Creation of Patient Videos

*Your Voice! Your Health!* was a research project that aimed to improve shared decision making (SDM) among clinicians and SGM racial and ethnic minority patients.^12–16^ The project captured the stories, views, preferences, and recommendations of these patients through audiotaped interviews and focus groups.^7,17,18^ After analyzing these data, our team invited five participants to be reinterviewed and share their stories through video. We created five 10- to 15-minute videos to capture a range of lived experiences and stories navigating health care, including a Latina lesbian gender nonconforming individual with a history of obesity/body image issues, an older African American gay man, an African American transfemme individual with chronic health conditions, a Latina transsexual woman, and an Asian American transmasculine individual who was a survivor of intimate partner violence (all self-defined identities). Coauthors Stephanie Bi, Kathryn E. Gunter, and Aviva G. Nathan chose 2- to 5-minute sections of these videos that focused on the educational objectives of our intersectionality teaching module (Appendices A–D). Due to time constraints, we did not show the Asian American transmasculine individual's video but have included it here as an optional video (Appendix E).

### Health Care Disparities: Equity and Advocacy

Health Care Disparities: Equity and Advocacy is the first course that first-year medical students at the Pritzker School of Medicine take, concurrently with human anatomy. Six weeks prior to this session, students received a 2-hour safe space training as a separate course module that introduced topics such as SGM terminology and the importance of the use of pronouns. However, essentially no prerequisite knowledge was necessary for our session, as the didactic presentation in the session reviewed key foundational material (Appendix F). The students had not taken any history-taking or clinical skills courses prior to our session.

### Teaching Module on Intersectionality of Sexual Orientation, Gender Identity, and Race/Ethnicity

A 30-minute didactic lecture, given by coauthor Scott C. Cook, a codirector of a national program to advance health equity, oriented students to concepts of SGM health care disparities, intersectionality, minority stress, and SDM. Coauthor Stephanie Bi, then a second-year Pritzker medical student, subsequently led the 1.5-hour facilitated discussion portion of the session. Stephanie Bi established ground rules to promote respect for others’ opinions and lived experiences. These were created by Monica B. Vela with feedback from prior students. Students viewed four video interviews of SGM POC describing their lives and health care narratives, each followed by moderated discussion about how compounded minority stress affects lived experiences and health, how multiple identities affect patient-provider communication, and how to improve SDM for SGM POC. Suggested discussion questions for each video can be found in Appendix G. Following the facilitated discussion, one of the video interviewees, DB, who had experience training clinicians in SGM issues, participated in a 30-minute question-and-answer session with the students. His video was shown, but no discussion was facilitated by coauthor Stephanie Bi. DB primarily focused on his experiences as an older African American gay man. He was compensated with an honorarium. Projection equipment and microphones were necessary to implement this session.

### Evaluation

Pre- and postsurveys (Appendix H) were sent to the students electronically via Google Forms. The presurvey was administered 7 days before the session, and the postsurvey was administered the same day the module was taught. The individual who sent out the surveys and then collected and deidentified the data was a research administrator within the University of Chicago external to our authorship team. Quantitative questions used Likert scales to query about self-assessed confidence in knowledge of SGM patients’ barriers, intersectionality, and communication with SGM patients, as well as general satisfaction with the session. The Likert items, ranging from *not at all confident* to *completely confident,* were assigned values from 1 to 5, respectively, which were used to calculate means and standard deviations. Qualitative questions asked for understanding of the term *intersectionality,* and on the postsurvey, respondents were asked to provide feedback on the strengths of the session and suggestions for improvement. We analyzed answers to the qualitative questions to inductively generate themes for course strengths and suggestions for improvement and to assess overall change in understanding of the definition of intersectionality. Comments made by even one participant were included as a theme if the response informed improvement of future sessions. Quantitative data were analyzed using the Wilcoxon rank sum test (unmatched pairs) to compare differences in self-reported knowledge and skills about SGM populations and intersectionality between pre- and postsession surveys. Data were analyzed using Stata 15. This study was deemed exempt by the University of Chicago Biological Sciences Division Institutional Review Board on August 9, 2018 (IRB18-1153).

## Results

Of the 89 total students in the class, 82 (92%) filled out the presurvey, and 83 (93%) filled out the postsurvey. On the postsurvey, one participant stated that they were not able to attend the session.

Student confidence in knowledge and skills about SGM and intersectionality improved from pre- to postsession (Table 1). These results are represented graphically in the Figure.

**Table 1.**
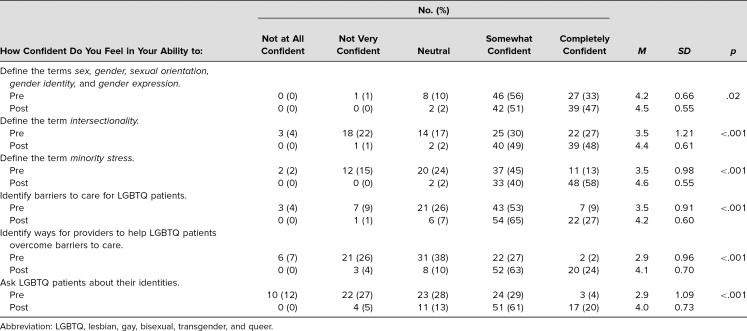
Changes in Confidence About LGBTQ and Intersectionality Knowledge and Skills Pre- and Postmodule

**Figure.**
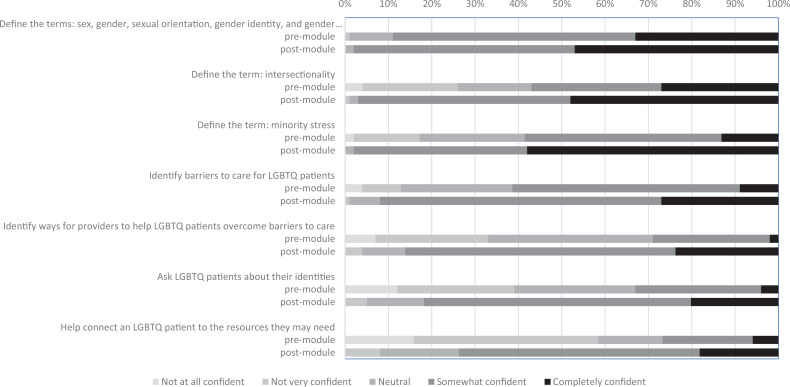
Changes in confidence about lesbian, gay, bisexual, transgender, and queer (LGBTQ) and intersectionality knowledge and skills.

### Understanding of Intersectionality

The students were asked on both the pre- and postsurveys, “What does intersectionality mean to you?” On the presurvey, most respondents were able to define intersectionality, but several respondents stated that they had not heard of the term and/or were unable to define it. For instance, one participant noted:

To be honest, this survey is the first time I have heard about the term of intersectionality. After searching it up, it seems like a way to describe how one's separate identities interact with each other. Perhaps this could be used to describe some of the identity conflicts that I face such as integrating my Chinese identity with my American identity? I am looking forward to learning more about this on Tuesday.

The terms *marginalized* and *minority* were used often. One participant responded, “The idea that individuals falling into multiple marginalized groups face greater challenges than simply the sum of challenges faced by each marginalized group individually.” Some answers, such as “Intersectionality means using my power/privilege to help minorities receive equity,” were related but slightly inaccurate.

On the postsurvey, all participants were able to define intersectionality, though some provided simplistic (“the combination of all my identities”) answers. One participant commented on the change in their understanding of intersectionality from before to after the session:

The definition of intersectionality remained the same for me, but compared to before, the word now has new intrinsic value that I should be aware of, along with examples of actual situations where multiple systems of oppression do limit and interfere with the care that patients receive and how we as health care workers serve.

### Strengths of the Session

Many participants emphasized the diversity of speakers (a medical center staff member, a near-peer student, and a community member) as a strength of the session. One comment includes, “I LOVED this class! This was the best session of the course. Having a researcher, a student facilitator and a community member who all worked together to deliver the session was a great model.” The use of a student facilitator was highlighted as a strength: “I thought [the student presenter] seemed very comfortable presenting and at times forgot that she was a student and not a resident or faculty member. It is amazing to me that she is only one year my superior.” The community member was also reported to be a critical addition to the workshop: “I thought that actually bringing in one of the ‘stars’ of the videos for a conversation was a great way to humanize and personalize what can be an abstract or theoretical topic else wise.” One participant commented on the lecture portion, “The first speaker… was also a great speaker and really fielded questions well.”

Several participants also remarked that they enjoyed being able to hear patient narratives firsthand through the video format, rather than just via statistics. One participant wrote:

I really appreciated hearing from individuals in the community grappling with these forces in society—forces that are systematically oppressive for their identity. It was a nice compliment [*sic*] to the more traditional lecture given prior, where we learned about pertinent statistics and models that characterize and quantify some of the experiences and consequences facing the community guest speakers.

Participants also noted the opportunity for interactive discussion as a strength of the session. One participant wrote:

The time [the student presenter] spent laying the ground rules for creating safe space was what I've been waiting for throughout the entire course. It worked: I heard comments and questions from my peers that made me proud, showcased our experience and curiosity, and allowed genuine space for learning.

Other strengths illustrated by the quotes listed below included the opportunity to learn tangible skills to aid SGM patients and an increased awareness of the impact of identities on SDM.

• Videos:
  ○ “I thought the videos and the following discussion were the strengths of the session. Hearing from patients and discussing how to navigate these issues, for example the standardization of pronouns, the sharing of knowledge with patients, meeting patients where they are and forming a partnership, was really helpful.”
  ○ “I really valued the videos with the accompanying discussion because it was helpful to have patients identifying as LGBTQ elaborate on the numerous barriers and micro-aggressions that they have previously faced when seeking health care. I learned about how I, as a future physician, could be more caring and well-informed when seeing patients who have intersectional identities.”
  ○ “The videos were the greatest strength as well as hearing from [the community member]. I've learned best about the topics in health care disparities when learning from actual patients, or people, who represent the affected population. For this reason, hearing these perspectives made the strongest impact on my experience with the session.”
• Interactive discussion:
  ○ “I wish all sessions were run like class today. The open discussion platform made it much easier for people to talk.”
• Student presenter:
  ○ “[The student] being a peer made everyone more receptive.”
  ○ “[The student] did an excellent job of encouraging our class to share and ask questions!”
  ○ “[The student] was an excellent moderator and led a really intellectual and insightful discussion.”
  ○ “THANK YOU, [student], for bringing us such a thoughtful, intentional, and rigorous learning experience.”
• Community member:
  ○ “Having [the community member] present to give his personal testimony and share his experience was so valuable and powerful. He was really charismatic and quite funny.”
  ○ “I loved how interactive it was and bringing in the actual person from the community was fantastic as he had a lot of input on how physicians can learn from their patients.”
• Tangible actions/skills:
  ○ “Talking through ways to phrase questions to patients to make them feel as safe and comfortable as possible is so important.”
  ○ “I thought the advice on how to approach intersectionality in clinic was particularly valuable. Learning how to phrase certain questions and help LGBT patients feel comfortable in the clinic space will make us all better physicians.”
  ○ “I also found it extremely valuable to discuss the tangible steps that we, as future physicians, can immediately take in caring for our patients and making each and every patient feel safe and comfortable in the care that individual is receiving.”
• Awareness of the role of identities in SDM:
  ○ “The session brought direct examples of people who were affected by preconceptions on gender identity and sexual orientation and how they actually felt (offended and hurt) that opened my eyes to the actual consequences of actions that may seem minor to some.”
  ○ “Hearing individuals speak about their experiences helped solidify that every action physicians make towards their patients is noted.”
• Inspiration:
  ○ “I was educated and inspired by this session by [the student], by [the community member], by [the faculty member], and it was hugely impactful for me to see people working in all these ways in the issues I want to devote my career towards.”

### Suggestions for Improvement of the Session

Several participants suggested including breakout sessions in small groups, rather than spending the entire time together as a 90-person class. For example, one participant stated:

I would have appreciated an opportunity to discuss in smaller groups, to further explorer [*sic*] the various concepts we were discussing in lecture, and also to continue brainstorm ways that we can affect change in our future careers. In small groups, I think we hear more voices from the class, something that I typically really value.

Some suggested other activities like role-play, “direct skill/toolbox demonstration for patient interaction,” and “some examples of ‘say this, not that’ or giving us practice simulations and pointers for how to handle difficult discussions.”

Some recommended that the lecture section be shortened and less repetitive. One participant commented, “I wish the lecture portion was shorter and there was more time for discussion, which I found more engaging and powerful.” Another felt that the “lecture felt rushed, and it seemed like he was brushing over many of his points when it wasn't exactly necessary.” There seemed to have been redundancy noted both within the session and with prior sessions. One participant noted:

[A] lot of the beginning of the session went over the same definitions and concepts that we had already gone over in the Safe Spaces training. Removing some of that redundancy and using the time to dive into more nuances of intersectional LGBTQ issues would have been great.

One student suggested, “Readings before class [would] have helped.”

In terms of the video-discussion portion of the session, some recommended a more nuanced discussion. One commented:

I think there were a lot of important points in the videos that should've been unpacked and weren't ever discussed. For instance, Aurora said so many things about how being lesbian has affected how she's viewed not [only] by the Latinx community but by her mother, and nobody brought it up.

Similarly, another participant commented, “I think the questions after the video blurbs should include more discussion-based questions instead of asking for comprehension of relating the videos back to the material discussed beforehand.”

A few participants also expressed a desire for a patient panel or, generally, more SGM POC voices represented. For instance, one participant commented, “It would have been great to have a whole panel of patients to speak to in order to hear even more perspectives.”

Other quotes listed below express desires for more practical content, a break in the session, information on LGBTQ children/adolescents, and reduced pausing of videos during the discussion.

• Small groups:
  ○ “The only thing I would have added is to have small groups after—not the ones that we have been doing, but truly small (4 to 5 people per group). We need more opportunities to talk to classmates one-on-one about these issues—often times, students voice really painful things for them or others that go ignored. Having truly small groups forces engagement.”
• More practical content:
  ○ “As a supplement to the workshop, it would be so cool if we could be given a short training for how to ensure we are using language that is welcoming and inclusive. Perhaps including some examples of ‘say this, not that’ or giving us practice simulations and pointers for how to handle difficult discussions.”
• Break in the session:
  ○ “I could have used a break intermixed with today's presentation. We went straight through for two and a half hours so I actually had to step out to grab coffee during the presentation but unfortunately missed some time to do so.”
  ○ “It would've been good to have a break in the middle just because the whole session felt pretty long.”
• Include children and adolescents:
  ○ “The content focused on LGBT adults, but children and adolescents could also be highlighted.”
• Show each video uninterrupted:
  ○ “I found the strategy of pausing in the middle of the videos to discuss a bit frustrating: since I thought they were very engaging and well-edited, I would have liked to have watched the whole thing and then discussed after the video was over for each specific interviewee.”

## Discussion

Medical education rarely focuses on the intersectional identities of patients. Our module teaches the intersectionality of sexual orientation/gender identity and race/ethnicity. It educates students about health disparities for SGM people, especially those of color, and how to communicate with them in a respectful, productive manner.

We created powerful patient videos that added moving human narrative to the statistics usually presented in health care disparities classes. Students thoroughly enjoyed the opportunity to discuss issues generated by the videos. Understanding patients’ identities requires personal introspection and vulnerability, and thus, respectfully sharing and listening to classmates’ perspectives can be healing and fruitful if done in a safe environment. The teaching team of a medical center staff member, near-peer student, and community member helped engage students by allowing research and lived experience to work synergistically.

Students recommended that future iterations of our session include small groups, role-play, a patient panel, deeper and more nuanced discussion of the videos, and a shorter, less redundant lecture. In Table 2, we have included questions to be considered when adapting this session to another institution. These questions will inform which, if any, of the students’ suggestions to incorporate.

**Table 2.**
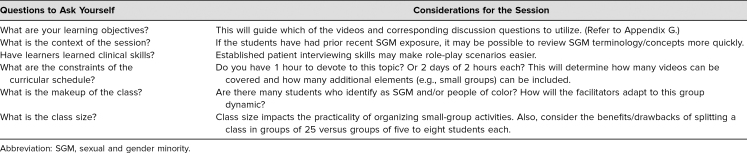
Tool for Adapting the Session to Your Own Institution

Small groups can be beneficial especially for learners who are uncomfortable with public speaking. Speaking up in our class of 90 can be daunting, much less in larger class sizes at other institutions. Small groups also allow each student more time to speak and may make them feel more comfortable sharing personal stories or controversial opinions. However, small groups can also be challenging because they can become unfocused. The success of a small group is often dependent on the capabilities of the facilitator and hence requires presession facilitator training, if enough committed and available facilitators can be identified.

We strove to balance depth and diversity of voices. Some of the comments recommended a patient panel to increase diversity of perspectives represented, while other comments suggested that our discussion of each video be more in-depth. These comments are somewhat at odds with each other given our time constraints. It would be beneficial to balance the simpler comprehension questions mentioned above by one participant with broader, more complex questions used to generate lively discussion. We have included a menu of discussion questions for each video in Appendix G. Other avenues for a patient panel may also be worth exploring, such as in a clinical skills course rather than a health care disparities course.

While the discussion questions serve to ignite conversation, we found it beneficial not to adhere to them too strictly. Listening to and commenting on contributions made by members of the class were more important. Some of the richest conversations emerged through students responding directly to one another, without much guidance from the facilitator. Flexibility in deviating from the discussion questions is therefore important to allow the conversation to flow organically.

Shortening the lecture has benefits and drawbacks. Especially in cultural competency medical education, students arrive at the session with different levels of experience and exposure to the subject at hand. Some students found the review of SGM terminology and concepts to be redundant. However, if we had not reviewed them in the lecture, some students might have been left behind. One student suggested assigning readings before the session. While there has been some promising research in favor of flipped classroom models for medical students,^19–21^ a major concern is that students might not complete the preassignment given competing time demands.

A limit to the generalizability of this session is that we had diverse racial, SGM, and other identities represented across the facilitators. This diverse representation added to the authenticity of the session. However, committed faculty and students of any identities can facilitate this session well, as the videos allow for SGM people of color to tell their stories even when there are few or none at an institution. A near-peer teacher for the video-discussion portion helped engage and inspire students in our workshop, but finding a near-peer presenter can be difficult given students’ interests and schedules. In addition, it should not be assumed that it is the responsibility of an SGM racial/ethnic minority student to teach the class about their community. It should also be noted that while individual experiences are important, SGM communities are diverse. Thus, no one individual's video should be considered to be representative of an entire community. We were fortunate that one of the video participants, DB, participated in an interactive question-and-answer portion of our session with students. A local SGM person of color could play a similar role, though it would not be the same as interacting with an actual video participant. Or the additional time could be used for more in-depth discussion of the videos (Appendices B and C) or allocated for one of the other options.

Our teaching module on intersectionality improved students’ knowledge of and confidence in caring for diverse patients. In future sessions, we aim to condense the lecture and potentially expand the SGM module of the Health Care Disparities: Equity and Advocacy course from one to two sessions to include small-group activities and/or a patient panel. Additionally, we would orient some of the discussion questions towards generating discussion rather than solely assessing comprehension of key concepts. Our curriculum is adaptable, and we hope it will be of value to all educators aiming to teach about intersectionality and improve the care of diverse patients such as SGM people of color.

## Appendices

Aurora Video.mp4Don Video.mp4Reyna Video.mp4Vita Video.mp4Sam Video.mp4Intersectionality Lecture.pptxSuggested Discussion Questions.docxPre- and Postsurveys.docx
All appendices are peer reviewed as integral parts of the Original Publication.
