# Supplementary material for: Teaching Intersectionality of Sexual Orientation, Gender Identity, and Race/Ethnicity in a Health Disparities Course
Source: MedEdPORTAL. 2020 Jul 31;16:10970. doi: 10.15766/mep_2374-8265.10970 (PMC7394350; doi:10.15766/mep_2374-8265.10970)
Supplement: Supplementary file 1 — Aurora Video.mp4Don Video.mp4Reyna Video.mp4Vita Video.mp4Sam Video.mp4Intersectionality Lecture.pptxSuggested Discussion Questions.docxPre- and Postsurveys.docx [file mep_2374-8265.10970-s001.zip › F. Intersectionality Lecture.pptx]

## Slide 1
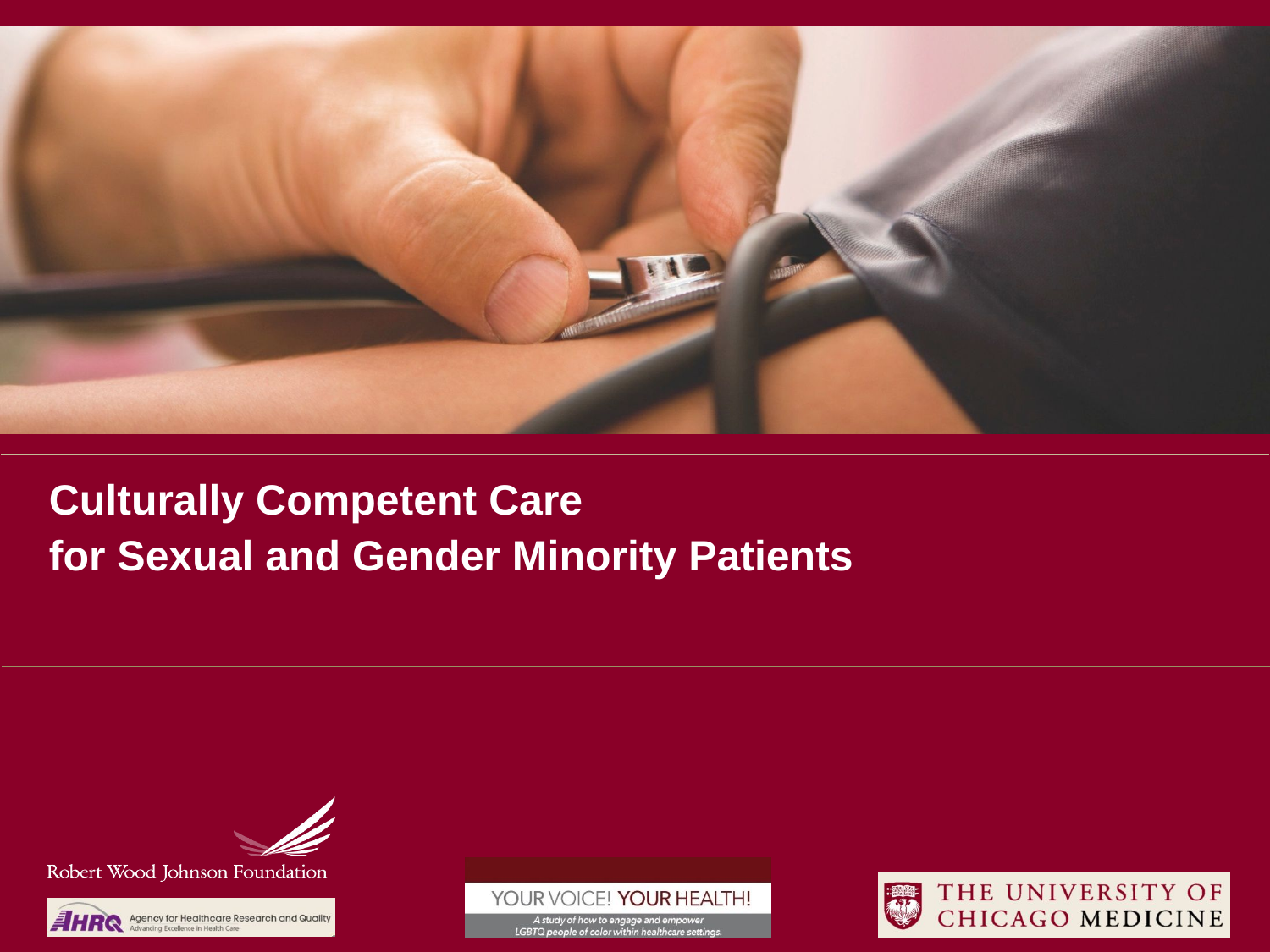

Culturally Competent Care
for Sexual and Gender Minority Patients

## Slide 2
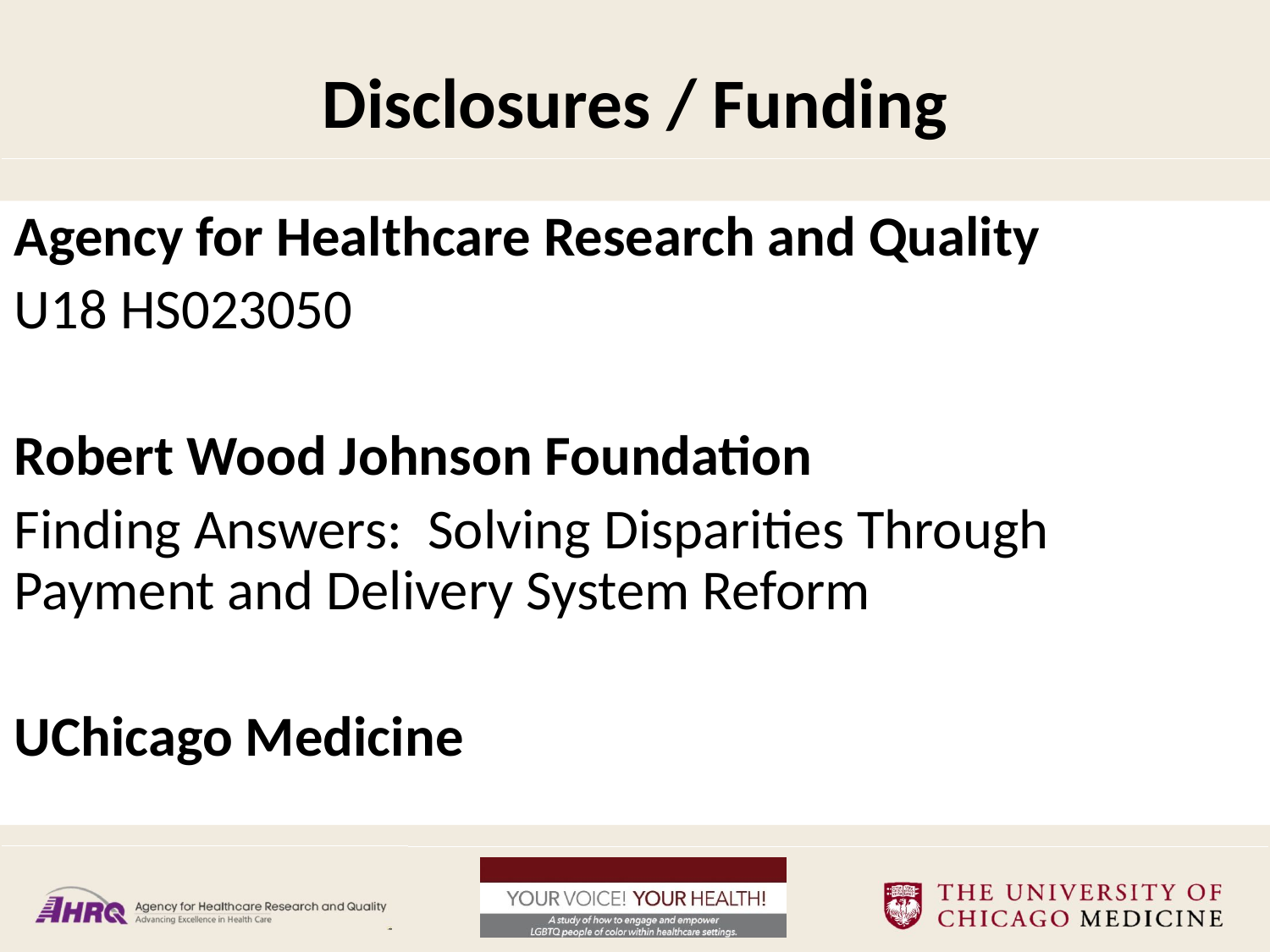

Disclosures / Funding
Agency for Healthcare Research and Quality
U18 HS023050
Robert Wood Johnson Foundation
Finding Answers: Solving Disparities Through Payment and Delivery System Reform
UChicago Medicine

## Slide 3
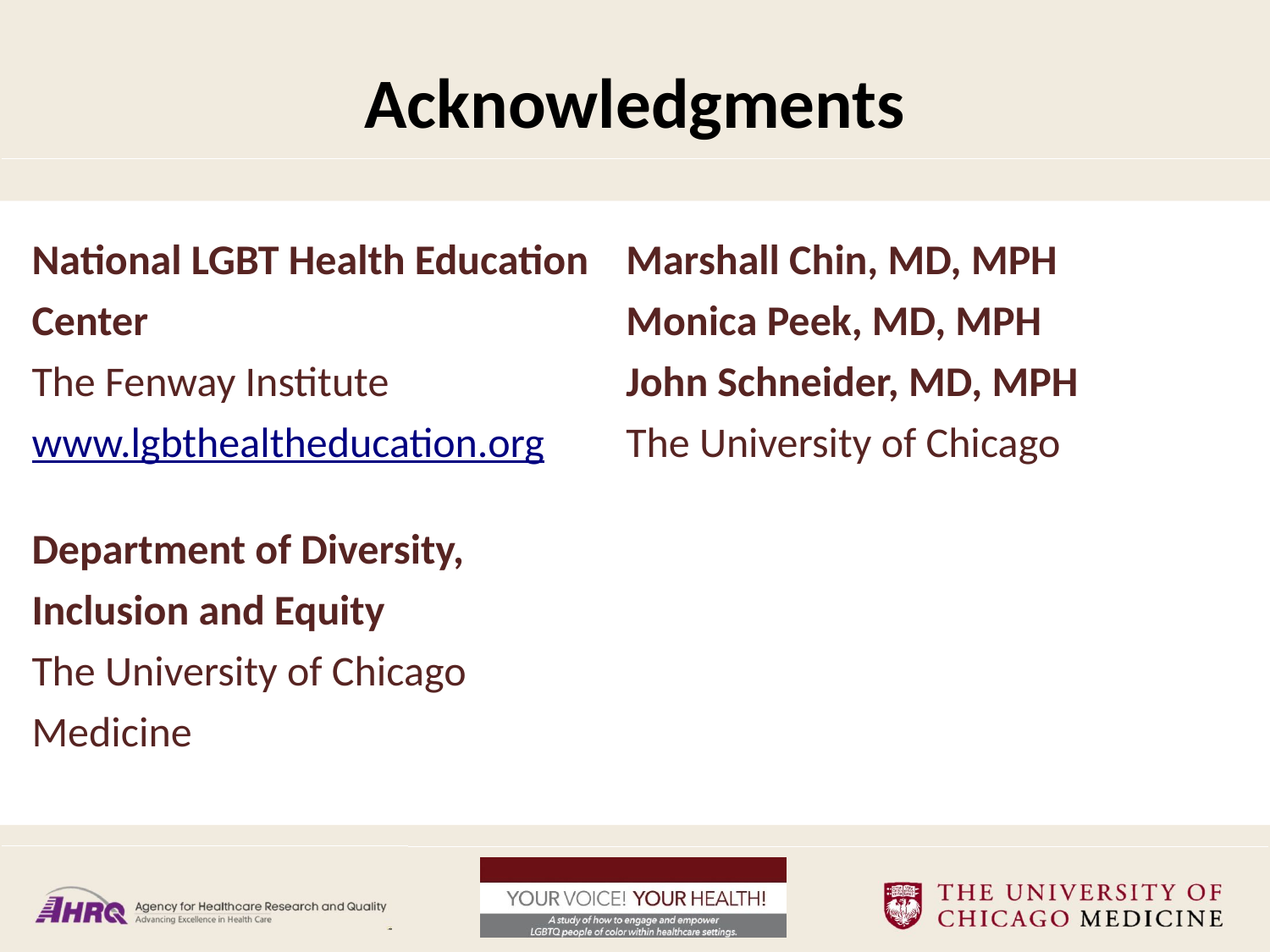

Acknowledgments
National LGBT Health Education Center
The Fenway Institute
www.lgbthealtheducation.org
Department of Diversity, Inclusion and Equity
The University of Chicago Medicine
Marshall Chin, MD, MPH
Monica Peek, MD, MPH
John Schneider, MD, MPH
The University of Chicago

## Slide 4
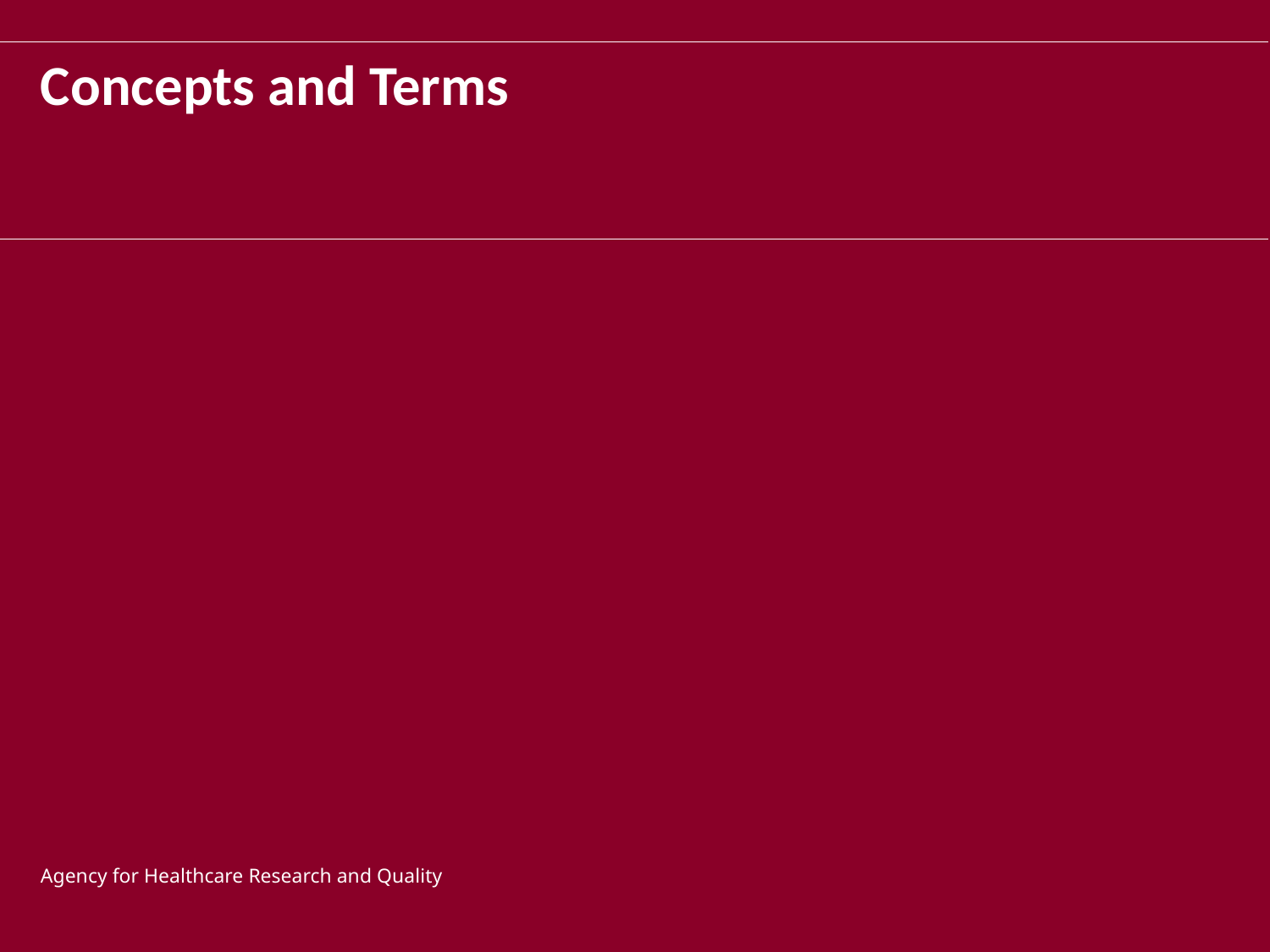

Concepts and Terms
Agency for Healthcare Research and Quality

## Slide 5
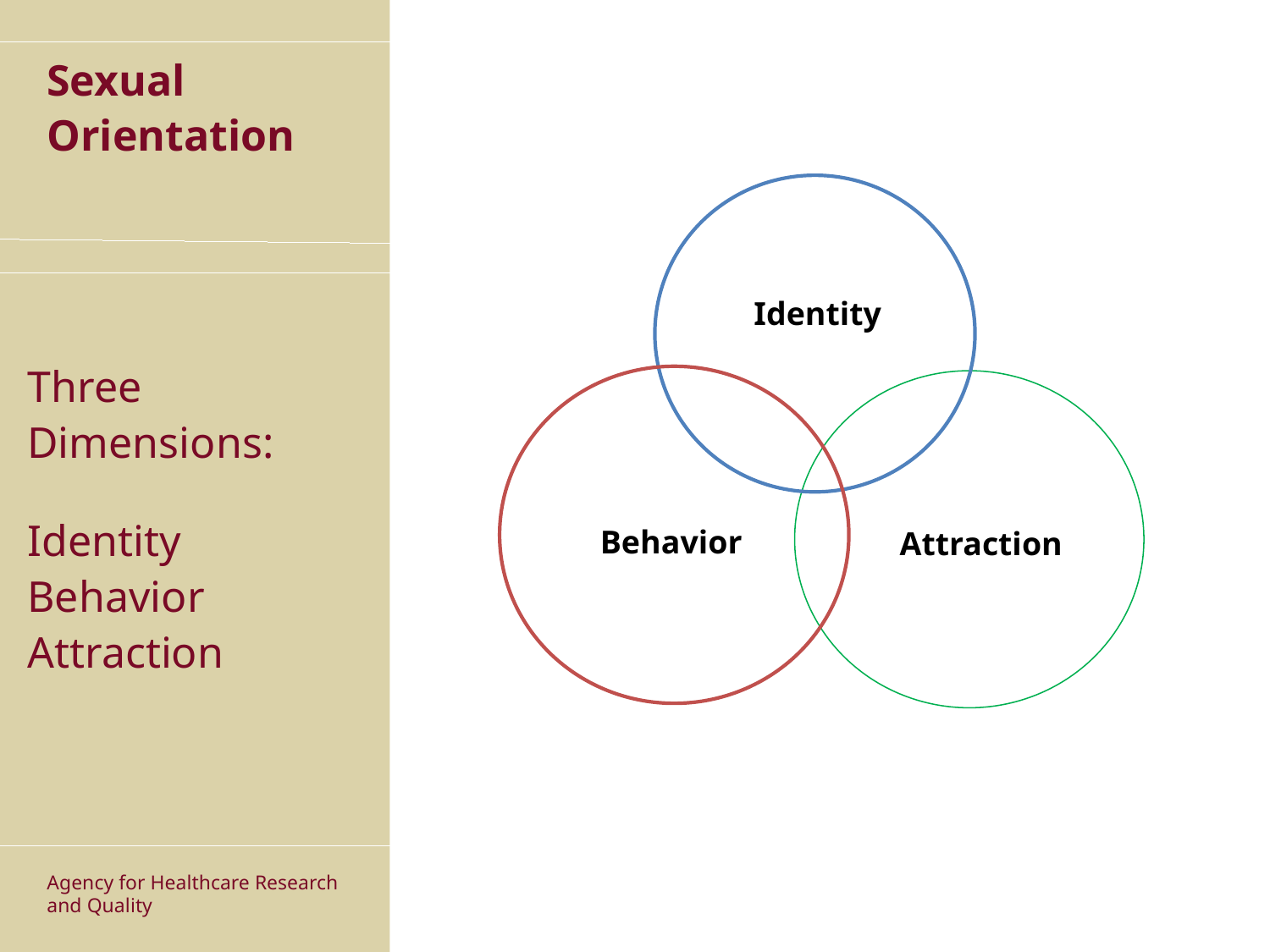

Sexual Orientation
Identity
Three Dimensions:
Identity Behavior Attraction
Behavior
Attraction
Agency for Healthcare Research
and Quality

## Slide 6
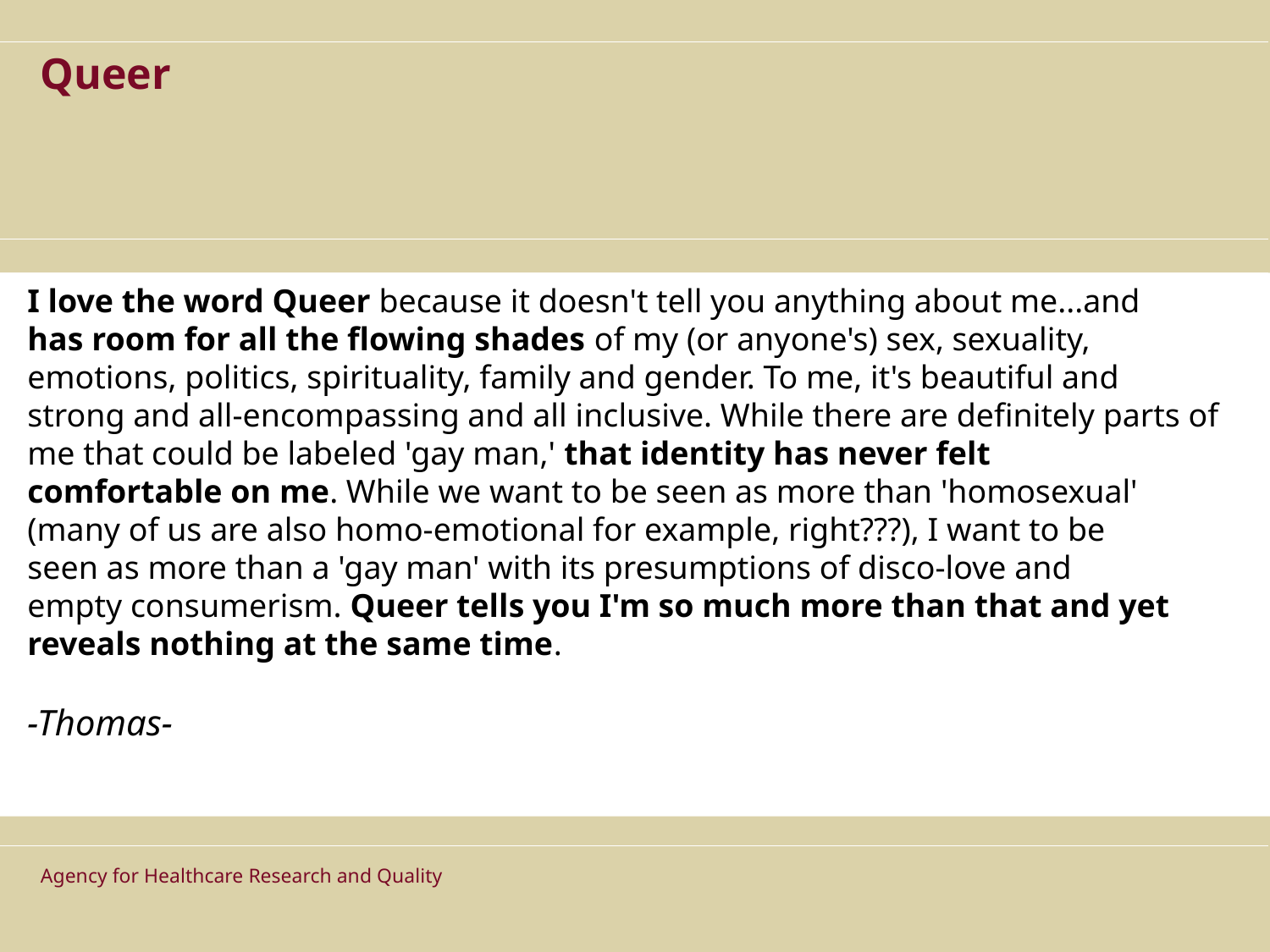

Queer
I love the word Queer because it doesn't tell you anything about me...and
has room for all the flowing shades of my (or anyone's) sex, sexuality,
emotions, politics, spirituality, family and gender. To me, it's beautiful and
strong and all-encompassing and all inclusive. While there are definitely parts of me that could be labeled 'gay man,' that identity has never felt
comfortable on me. While we want to be seen as more than 'homosexual'
(many of us are also homo-emotional for example, right???), I want to be
seen as more than a 'gay man' with its presumptions of disco-love and
empty consumerism. Queer tells you I'm so much more than that and yet
reveals nothing at the same time.
-Thomas-
Agency for Healthcare Research and Quality

## Slide 7
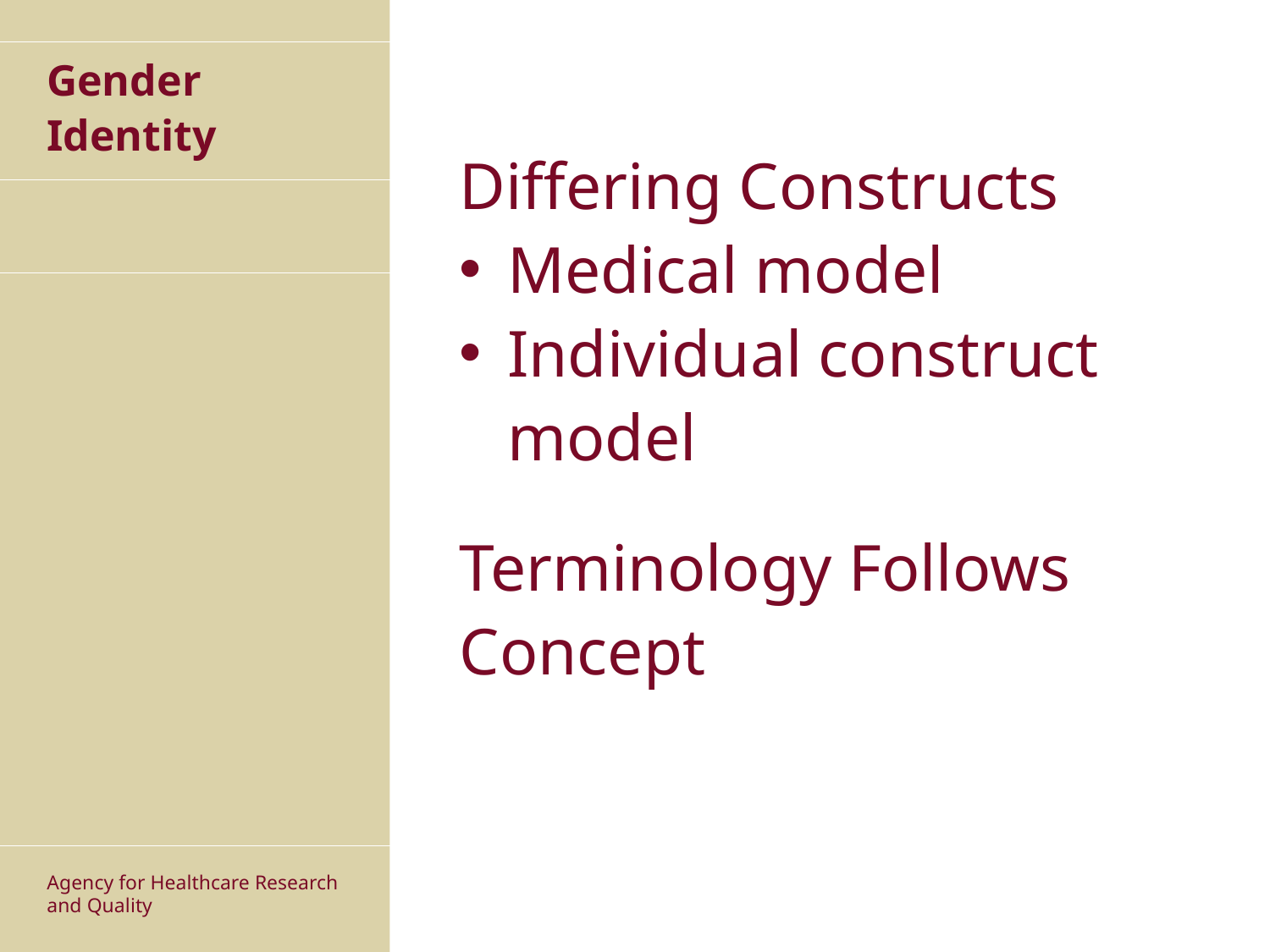

Gender Identity
Differing Constructs
Medical model
Individual construct model
Terminology Follows Concept
Agency for Healthcare Research
and Quality

## Slide 8
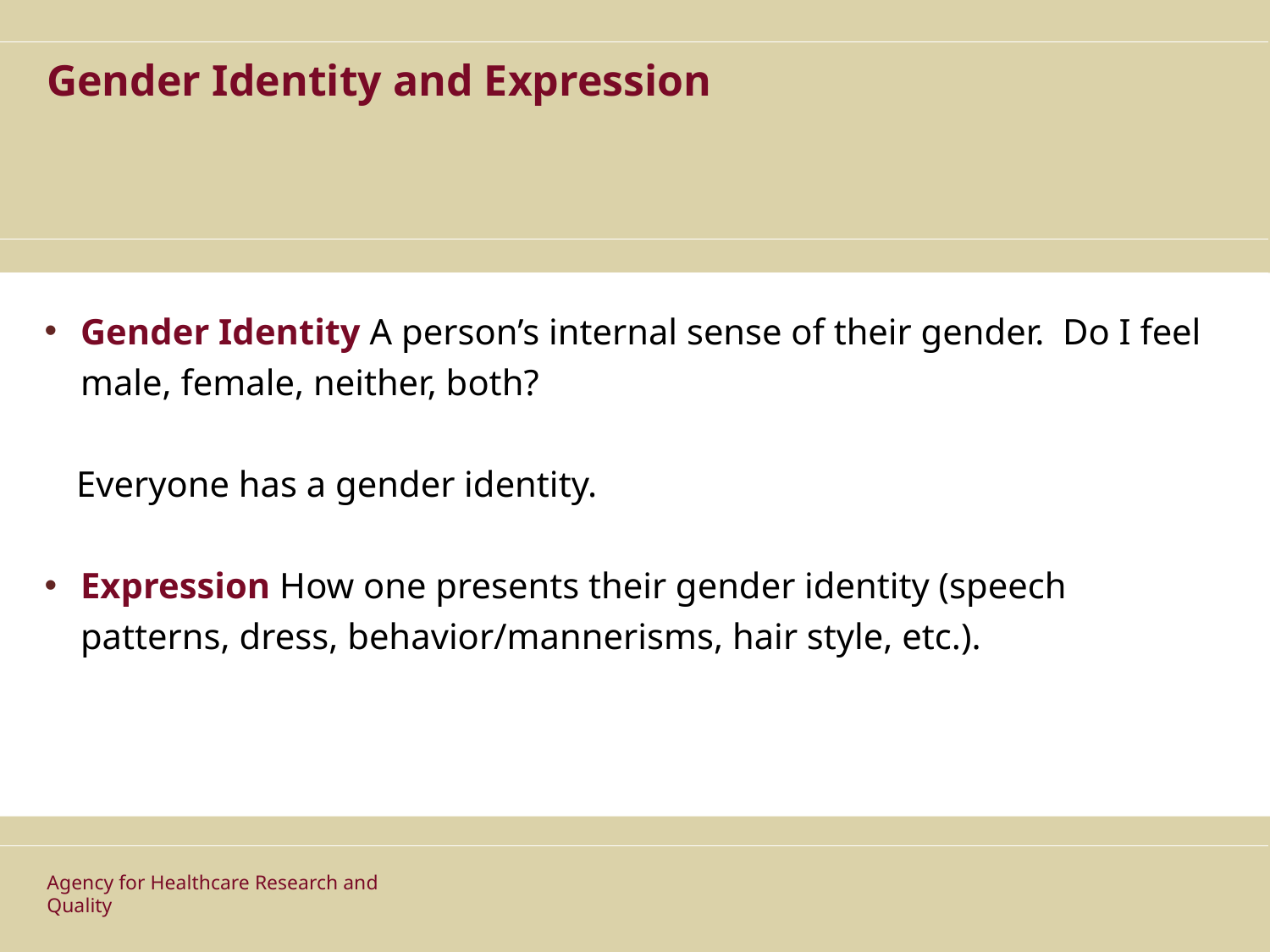

Gender Identity and Expression
Gender Identity A person’s internal sense of their gender. Do I feel male, female, neither, both?
Everyone has a gender identity.
Expression How one presents their gender identity (speech patterns, dress, behavior/mannerisms, hair style, etc.).
Agency for Healthcare Research and Quality

## Slide 9
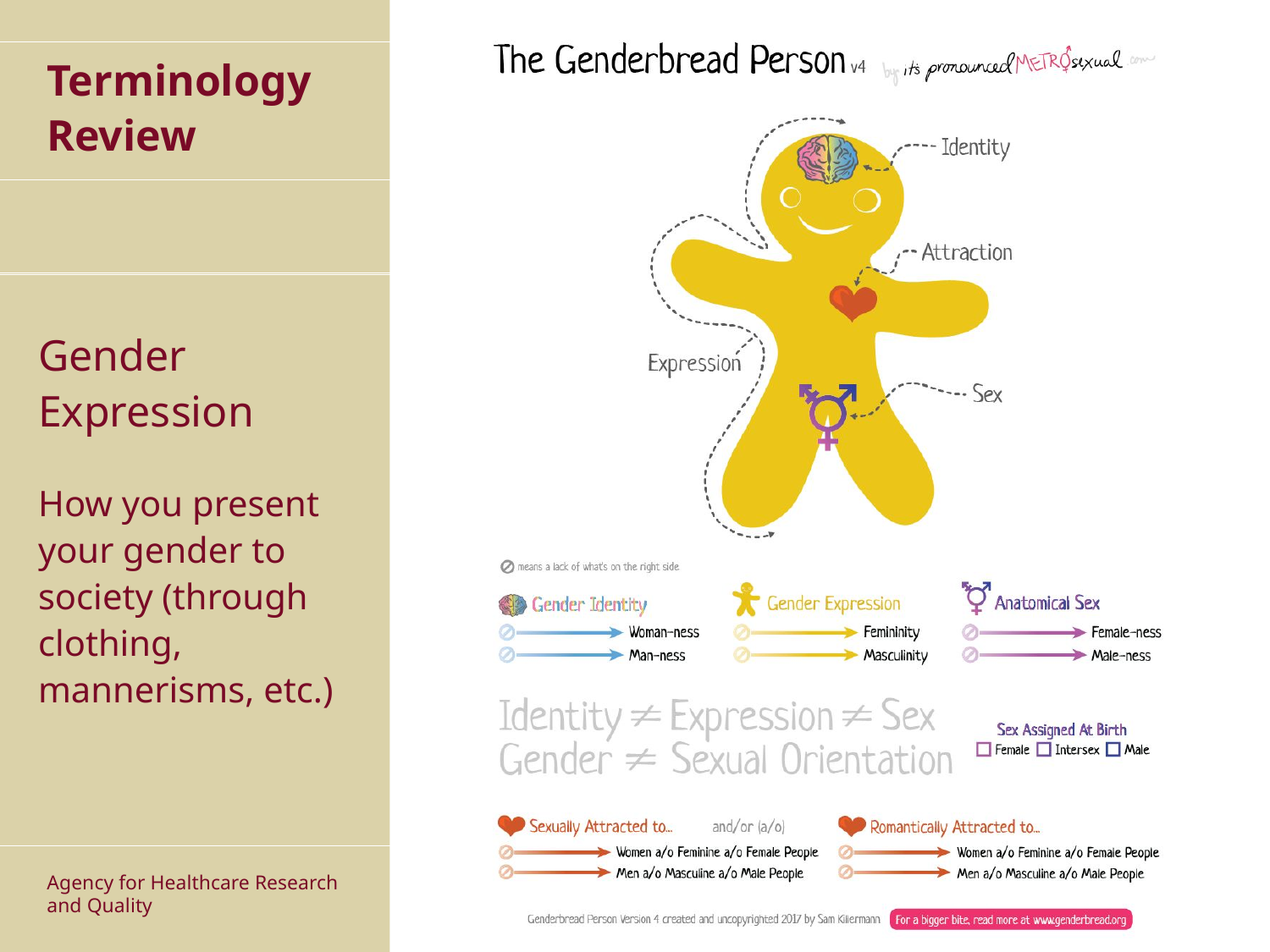

Terminology Review
Gender Expression
How you present your gender to society (through clothing, mannerisms, etc.)
Agency for Healthcare Research
and Quality

## Slide 10
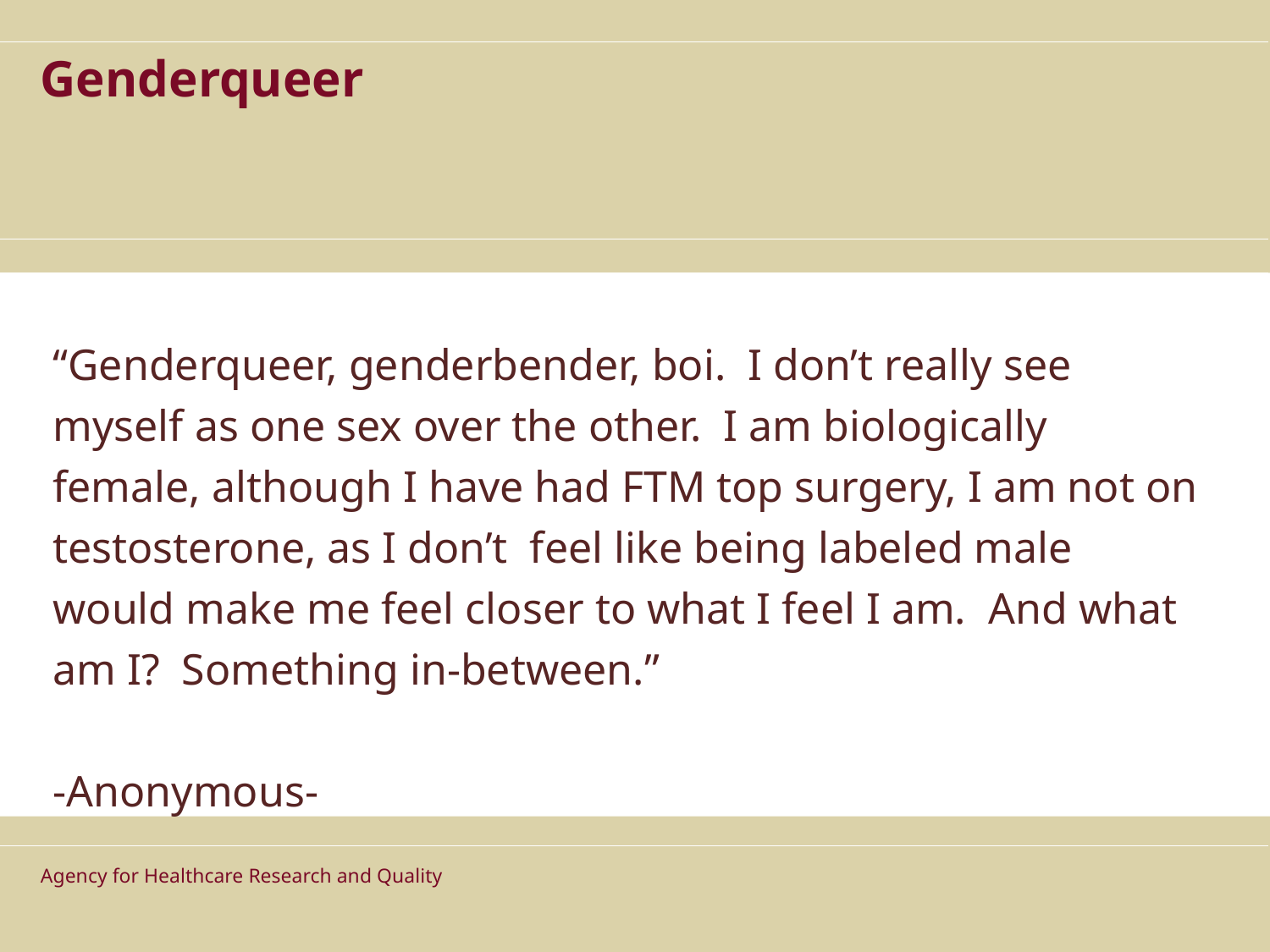

Genderqueer
“Genderqueer, genderbender, boi. I don’t really see myself as one sex over the other. I am biologically female, although I have had FTM top surgery, I am not on testosterone, as I don’t feel like being labeled male would make me feel closer to what I feel I am. And what am I? Something in-between.”
-Anonymous-
Agency for Healthcare Research and Quality

## Slide 11
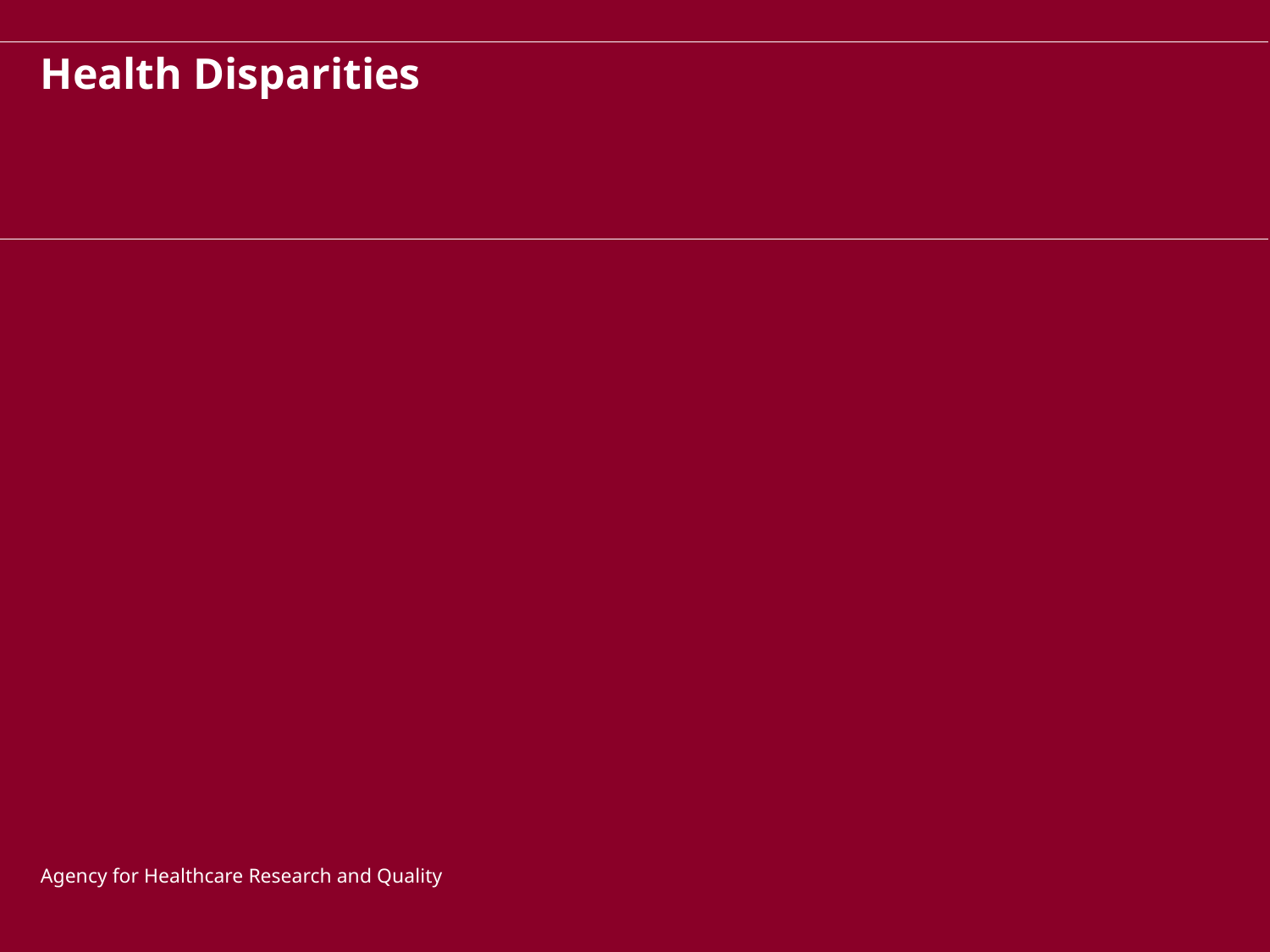

Health Disparities
Agency for Healthcare Research and Quality

## Slide 12
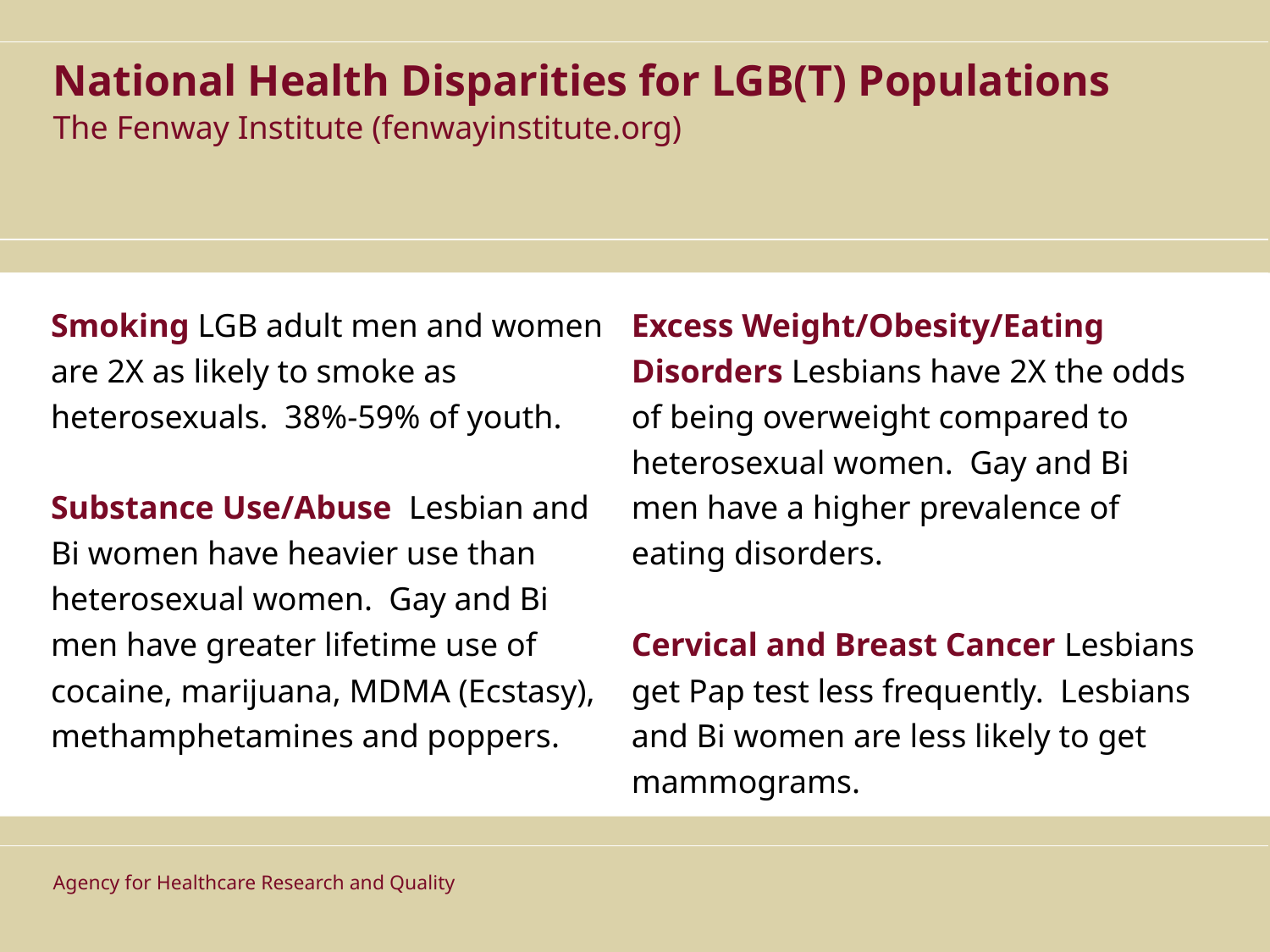

National Health Disparities for LGB(T) Populations
The Fenway Institute (fenwayinstitute.org)
Smoking LGB adult men and women are 2X as likely to smoke as heterosexuals. 38%-59% of youth.
Substance Use/Abuse Lesbian and Bi women have heavier use than heterosexual women. Gay and Bi men have greater lifetime use of cocaine, marijuana, MDMA (Ecstasy), methamphetamines and poppers.
Excess Weight/Obesity/Eating Disorders Lesbians have 2X the odds of being overweight compared to heterosexual women. Gay and Bi men have a higher prevalence of eating disorders.
Cervical and Breast Cancer Lesbians get Pap test less frequently. Lesbians and Bi women are less likely to get mammograms.
Agency for Healthcare Research and Quality

## Slide 13
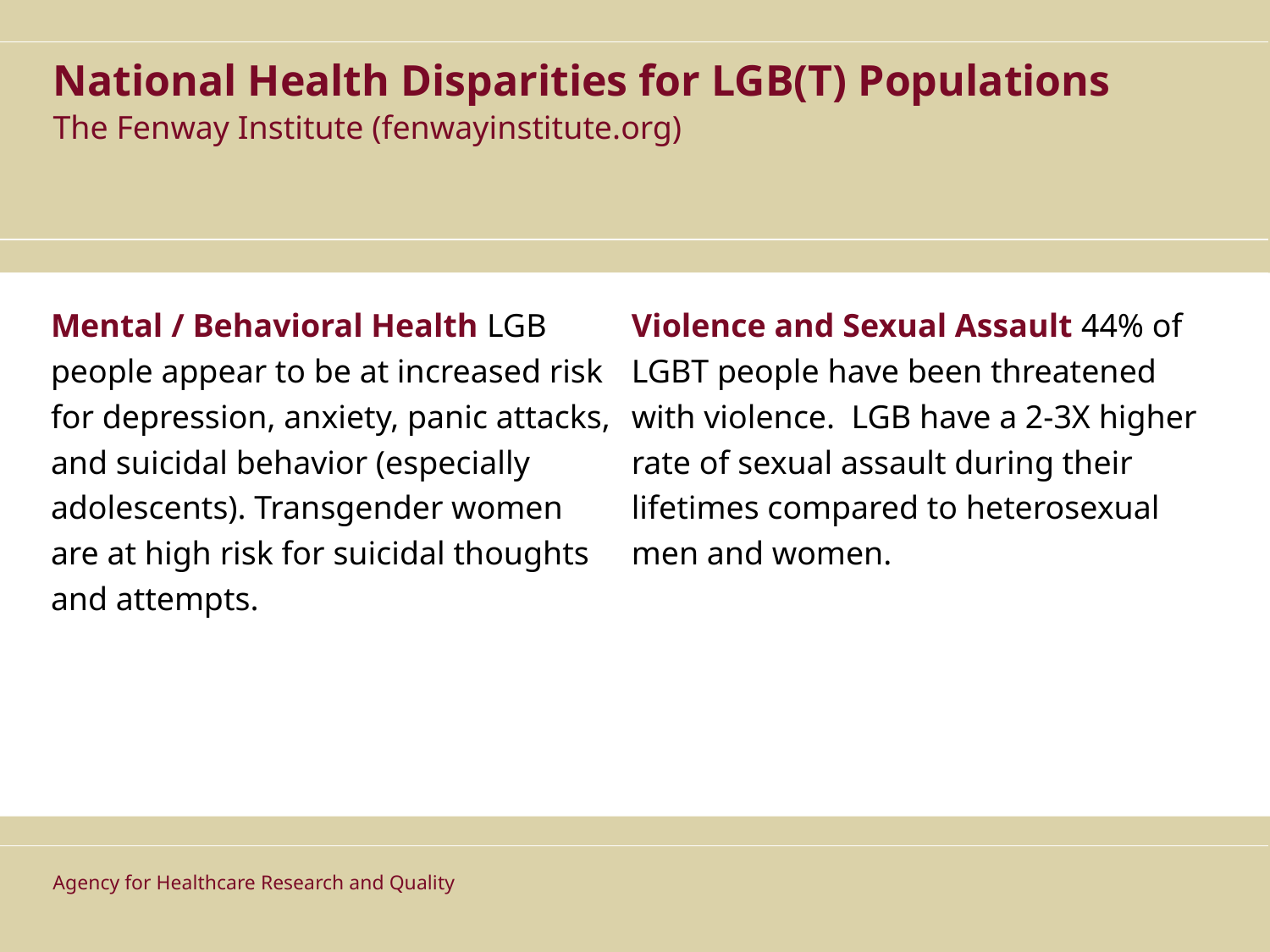

National Health Disparities for LGB(T) Populations
The Fenway Institute (fenwayinstitute.org)
Mental / Behavioral Health LGB people appear to be at increased risk for depression, anxiety, panic attacks, and suicidal behavior (especially adolescents). Transgender women are at high risk for suicidal thoughts and attempts.
Violence and Sexual Assault 44% of LGBT people have been threatened with violence. LGB have a 2-3X higher rate of sexual assault during their lifetimes compared to heterosexual men and women.
Agency for Healthcare Research and Quality

## Slide 14
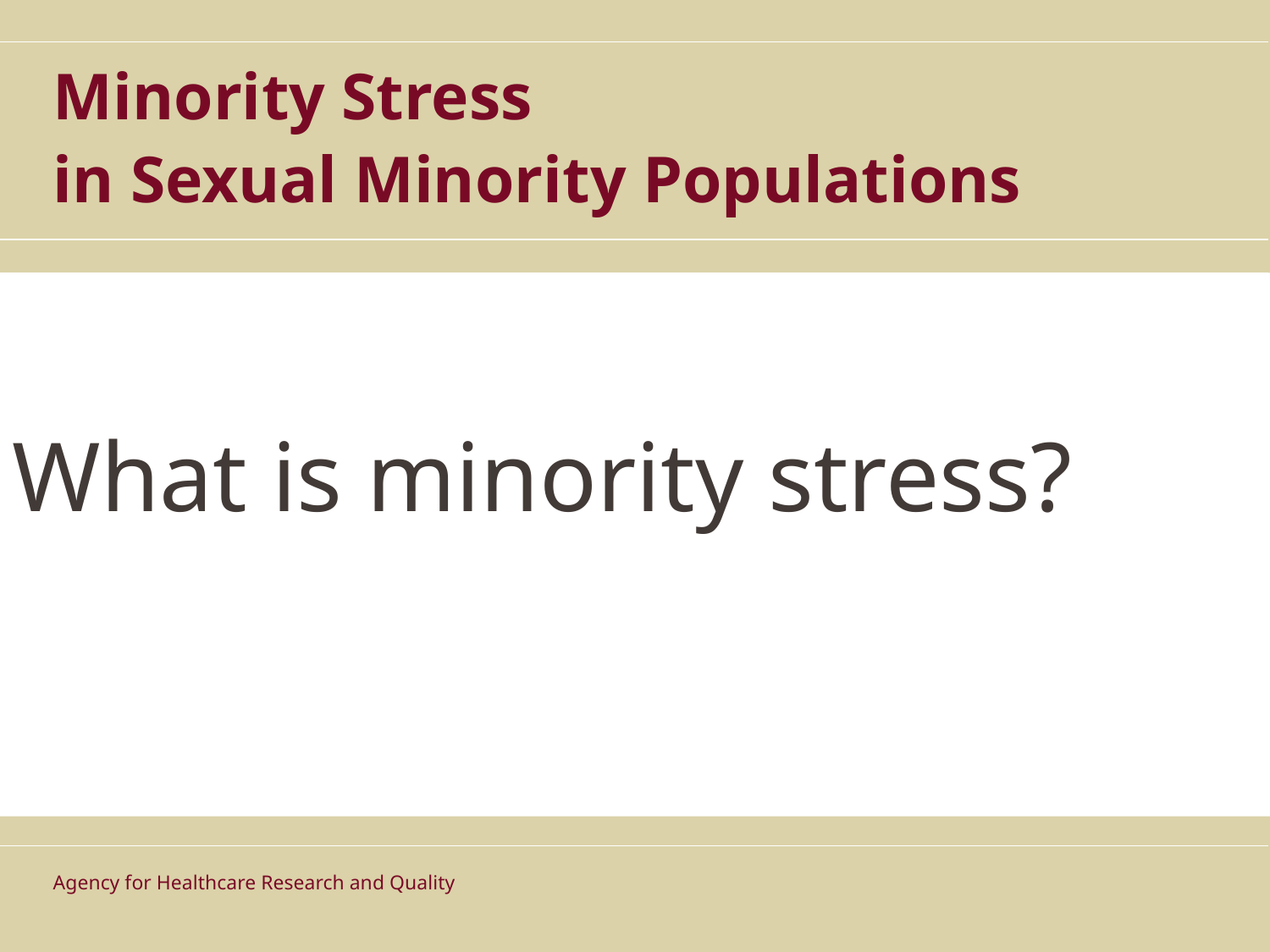

Minority Stress
in Sexual Minority Populations
What is minority stress?
Agency for Healthcare Research and Quality

## Slide 15
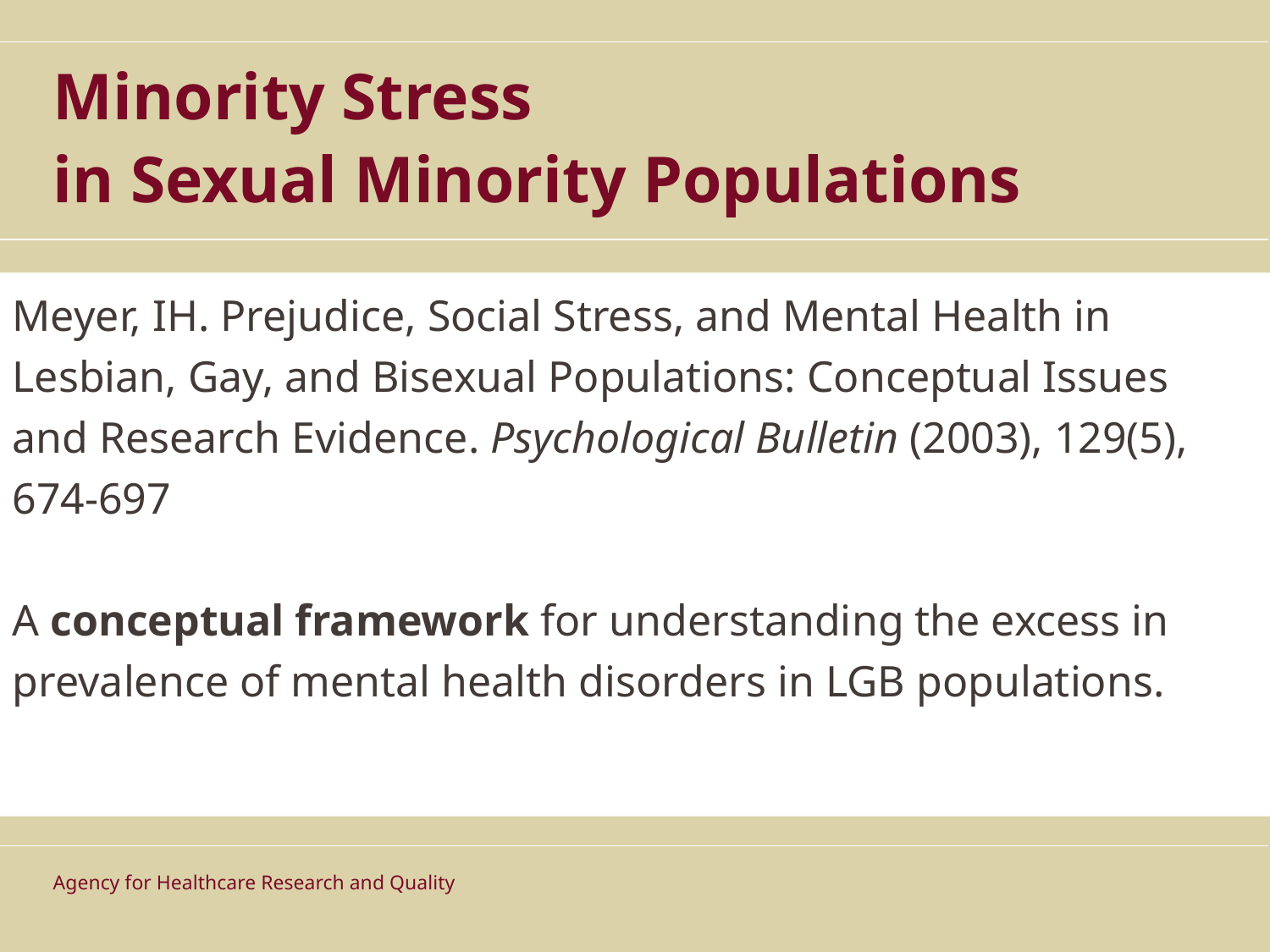

Minority Stress
in Sexual Minority Populations
Meyer, IH. Prejudice, Social Stress, and Mental Health in Lesbian, Gay, and Bisexual Populations: Conceptual Issues and Research Evidence. Psychological Bulletin (2003), 129(5), 674-697
A conceptual framework for understanding the excess in prevalence of mental health disorders in LGB populations.
Agency for Healthcare Research and Quality

## Slide 16
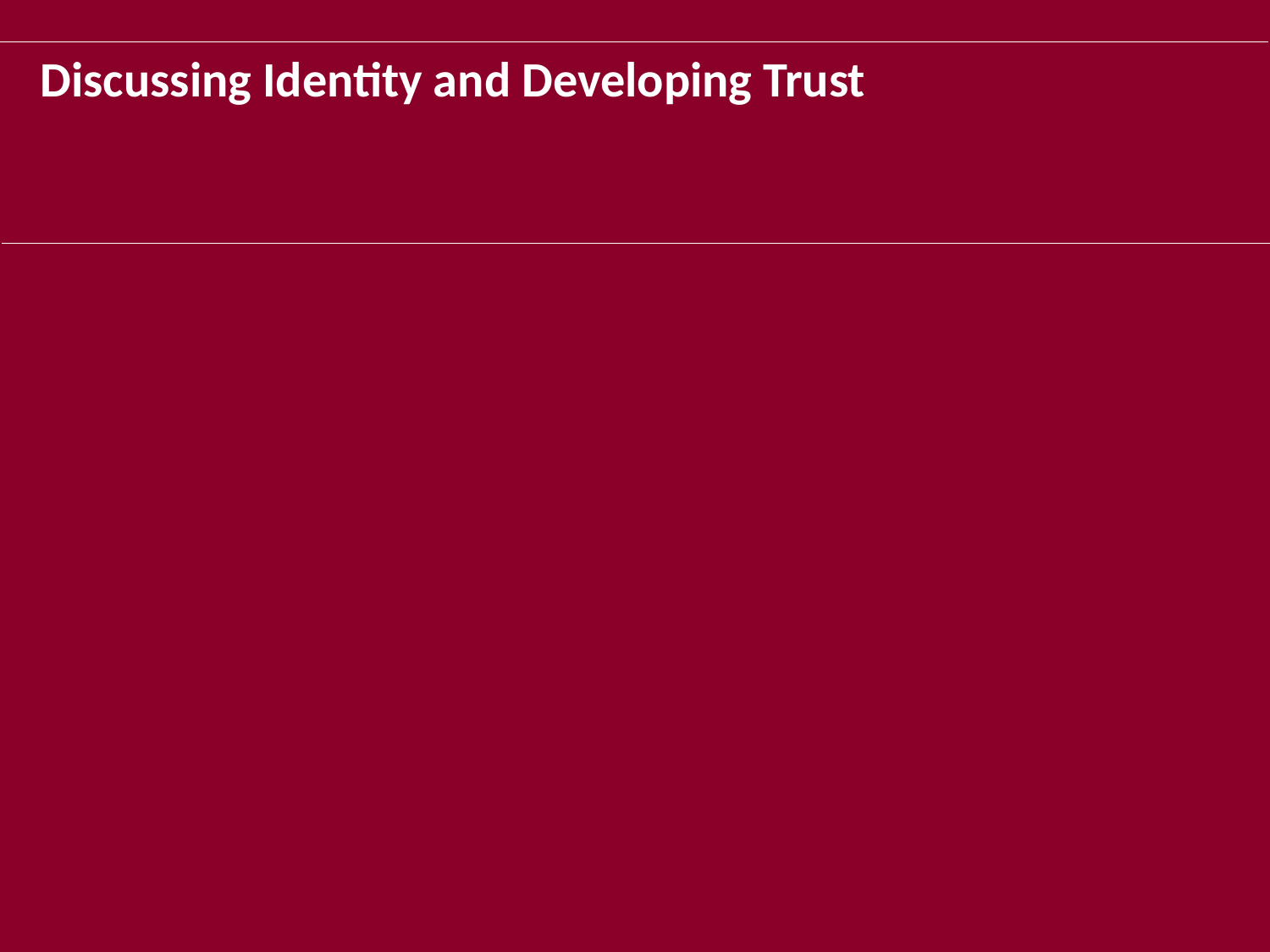

Discussing Identity and Developing Trust

## Slide 17
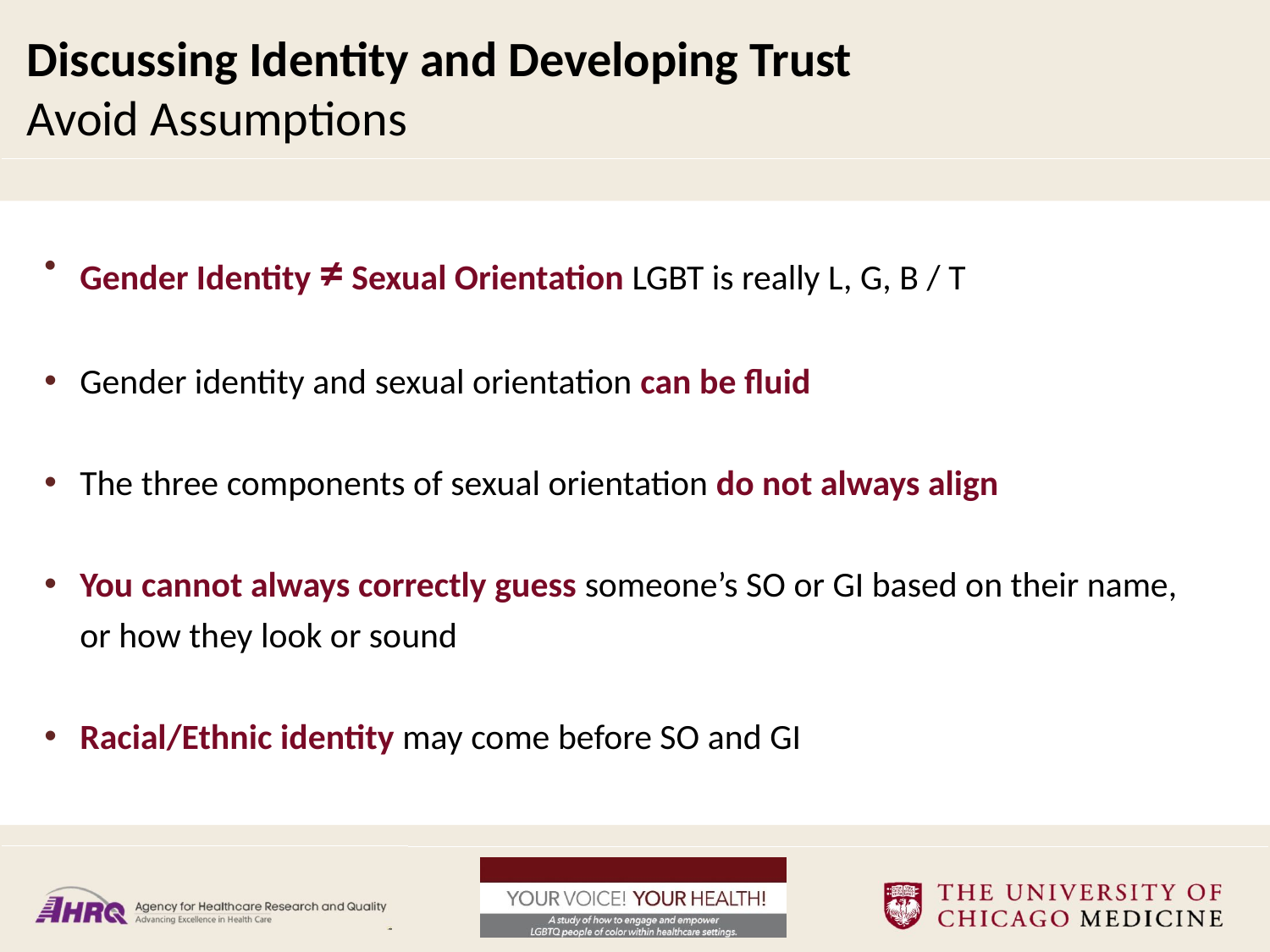

Discussing Identity and Developing Trust
Avoid Assumptions
Gender Identity ≠ Sexual Orientation LGBT is really L, G, B / T
Gender identity and sexual orientation can be fluid
The three components of sexual orientation do not always align
You cannot always correctly guess someone’s SO or GI based on their name, or how they look or sound
Racial/Ethnic identity may come before SO and GI

## Slide 18
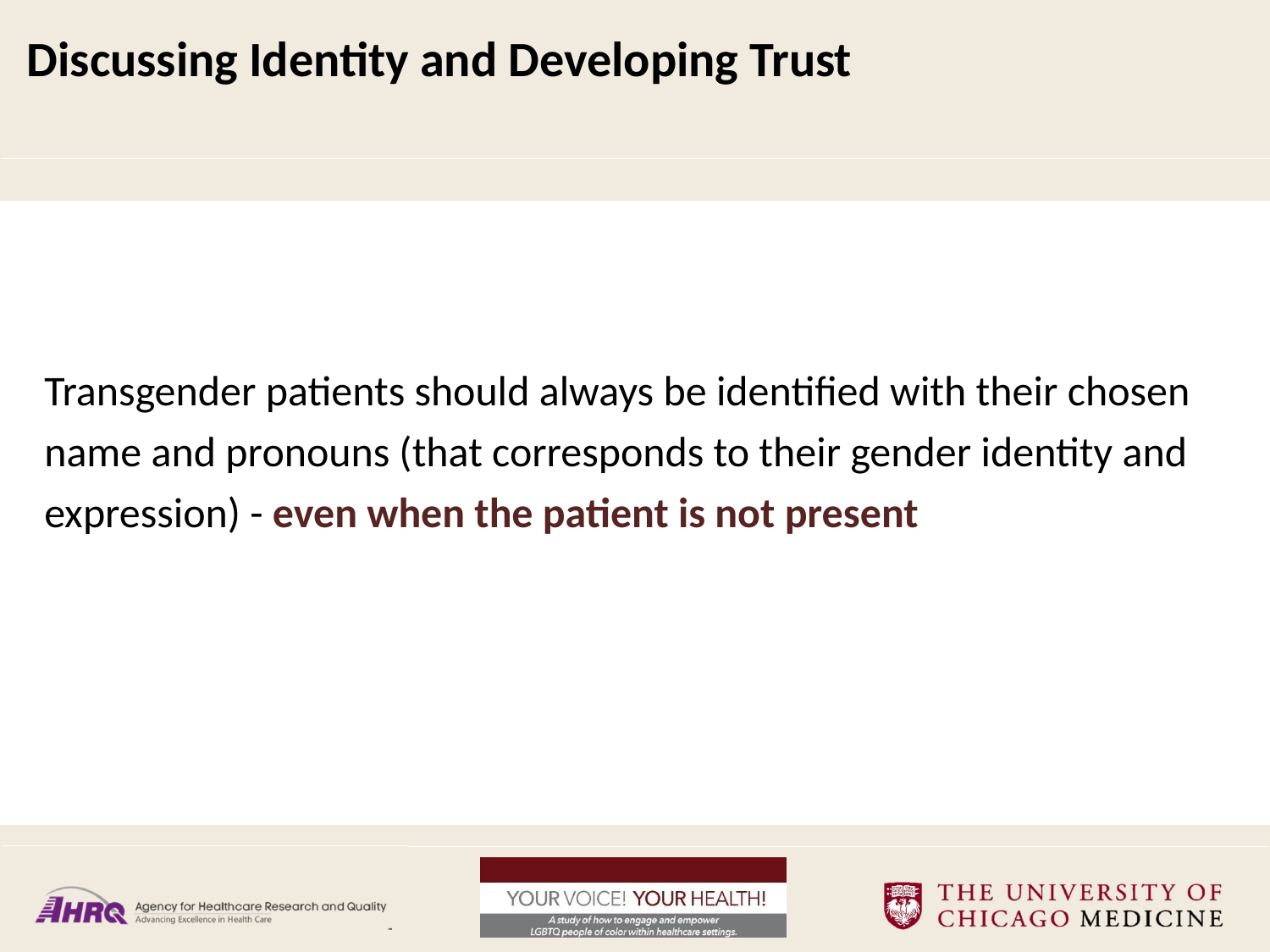

Discussing Identity and Developing Trust
Transgender patients should always be identified with their chosen name and pronouns (that corresponds to their gender identity and expression) - even when the patient is not present

## Slide 19
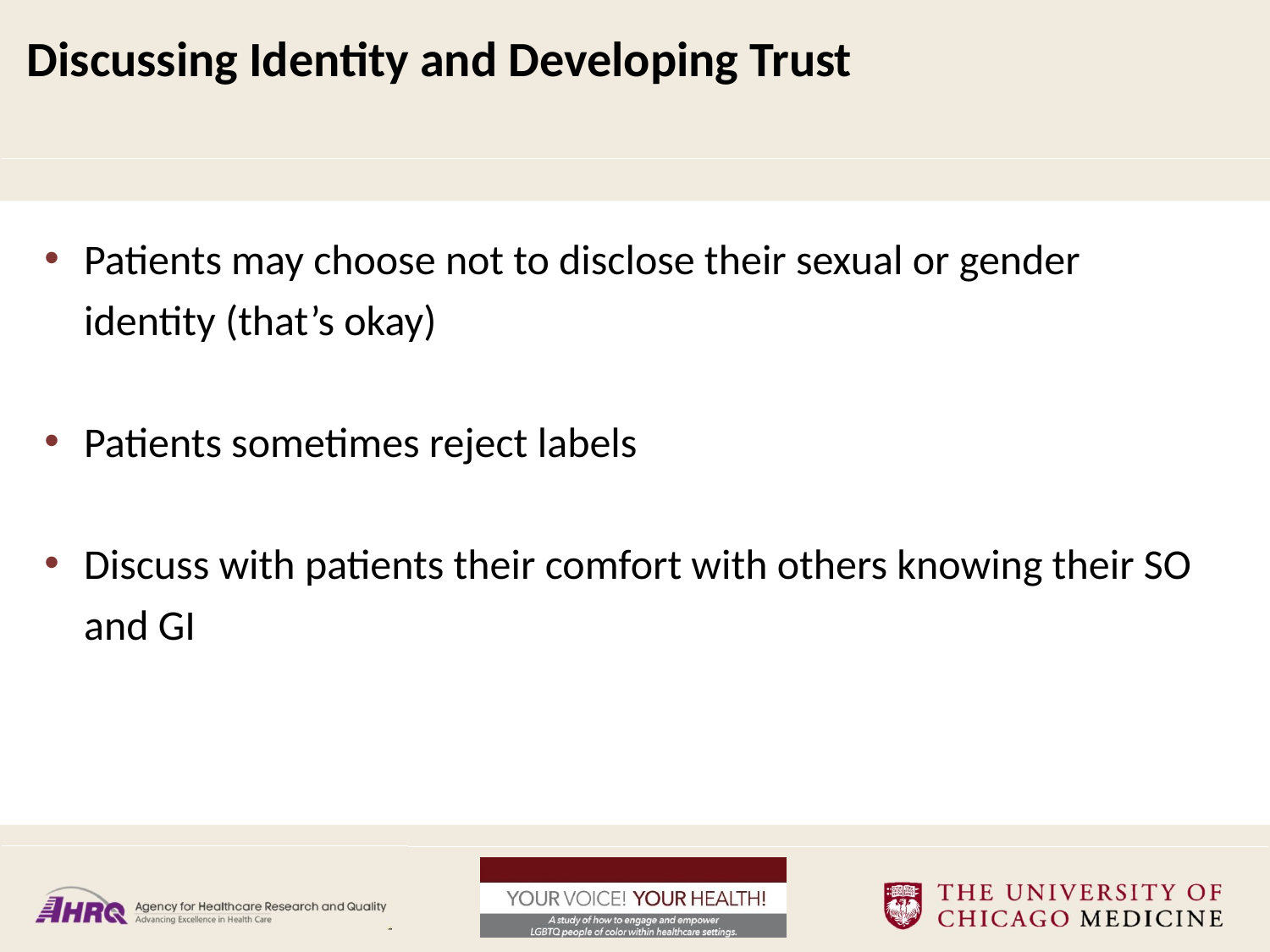

Discussing Identity and Developing Trust
Patients may choose not to disclose their sexual or gender identity (that’s okay)
Patients sometimes reject labels
Discuss with patients their comfort with others knowing their SO and GI

## Slide 20
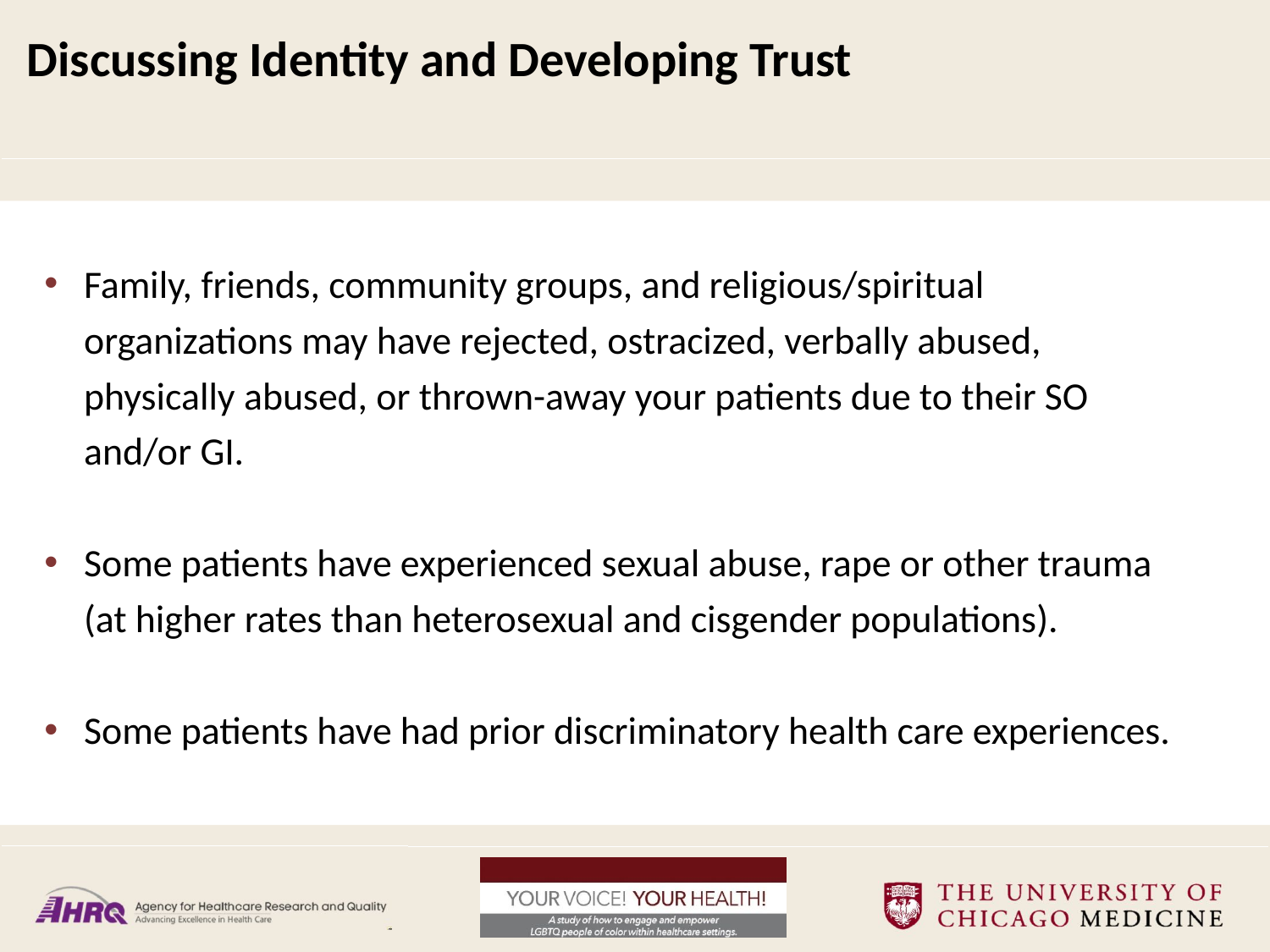

Discussing Identity and Developing Trust
Family, friends, community groups, and religious/spiritual organizations may have rejected, ostracized, verbally abused, physically abused, or thrown-away your patients due to their SO and/or GI.
Some patients have experienced sexual abuse, rape or other trauma (at higher rates than heterosexual and cisgender populations).
Some patients have had prior discriminatory health care experiences.

## Slide 21
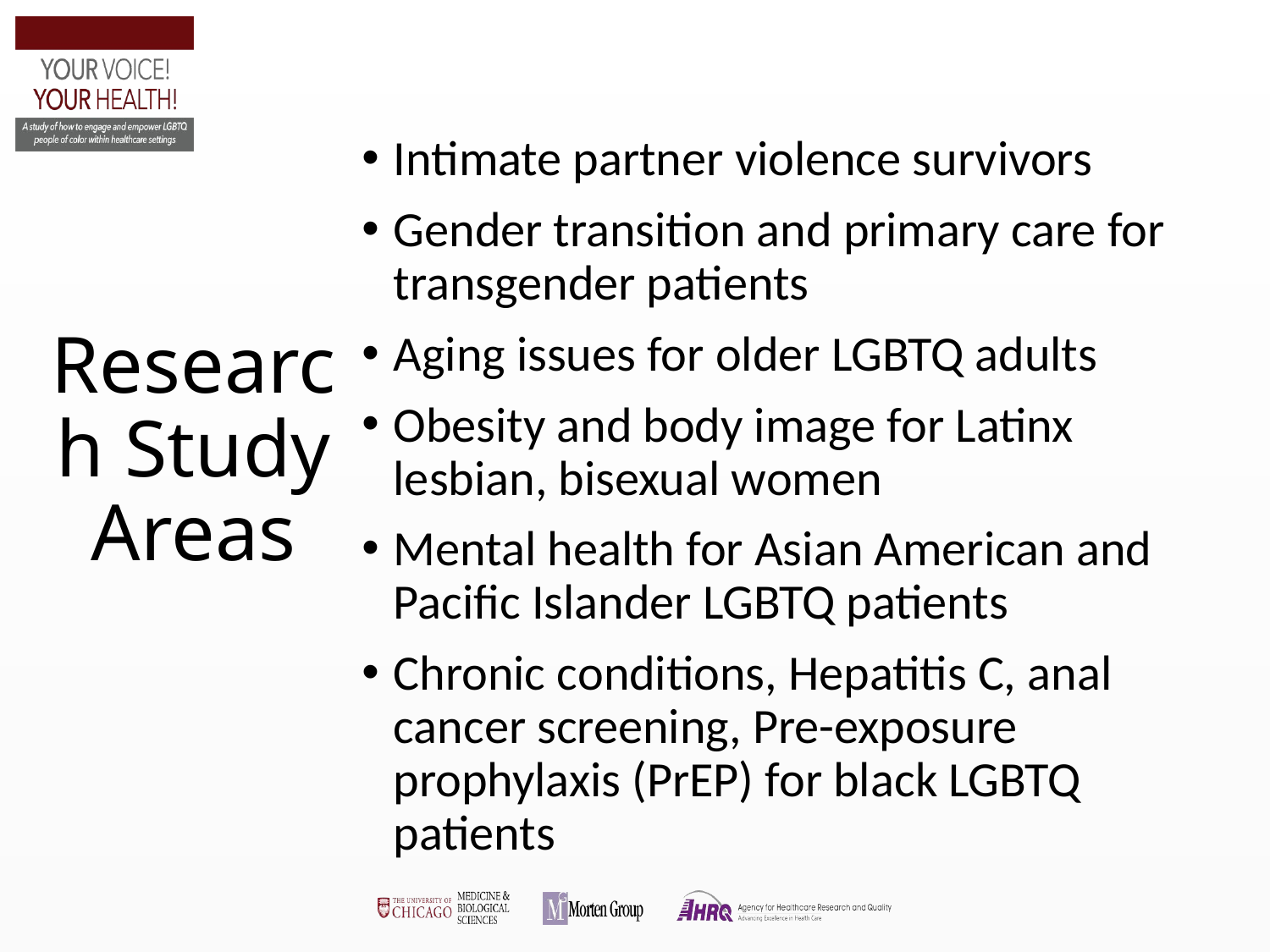

Intimate partner violence survivors
Gender transition and primary care for transgender patients
Aging issues for older LGBTQ adults
Obesity and body image for Latinx lesbian, bisexual women
Mental health for Asian American and Pacific Islander LGBTQ patients
Chronic conditions, Hepatitis C, anal cancer screening, Pre-exposure prophylaxis (PrEP) for black LGBTQ patients
Research Study Areas

## Slide 22
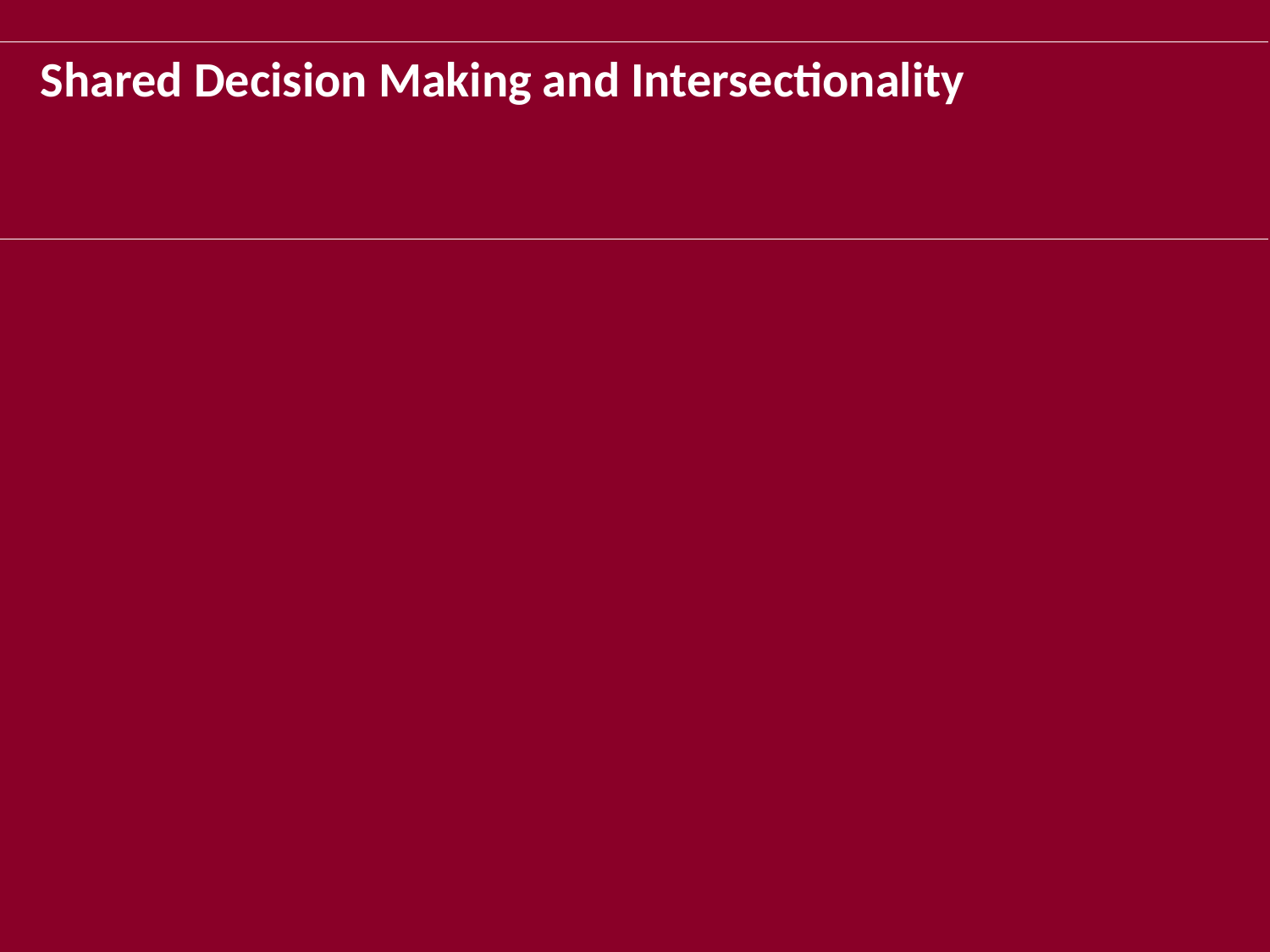

Shared Decision Making and Intersectionality

## Slide 23
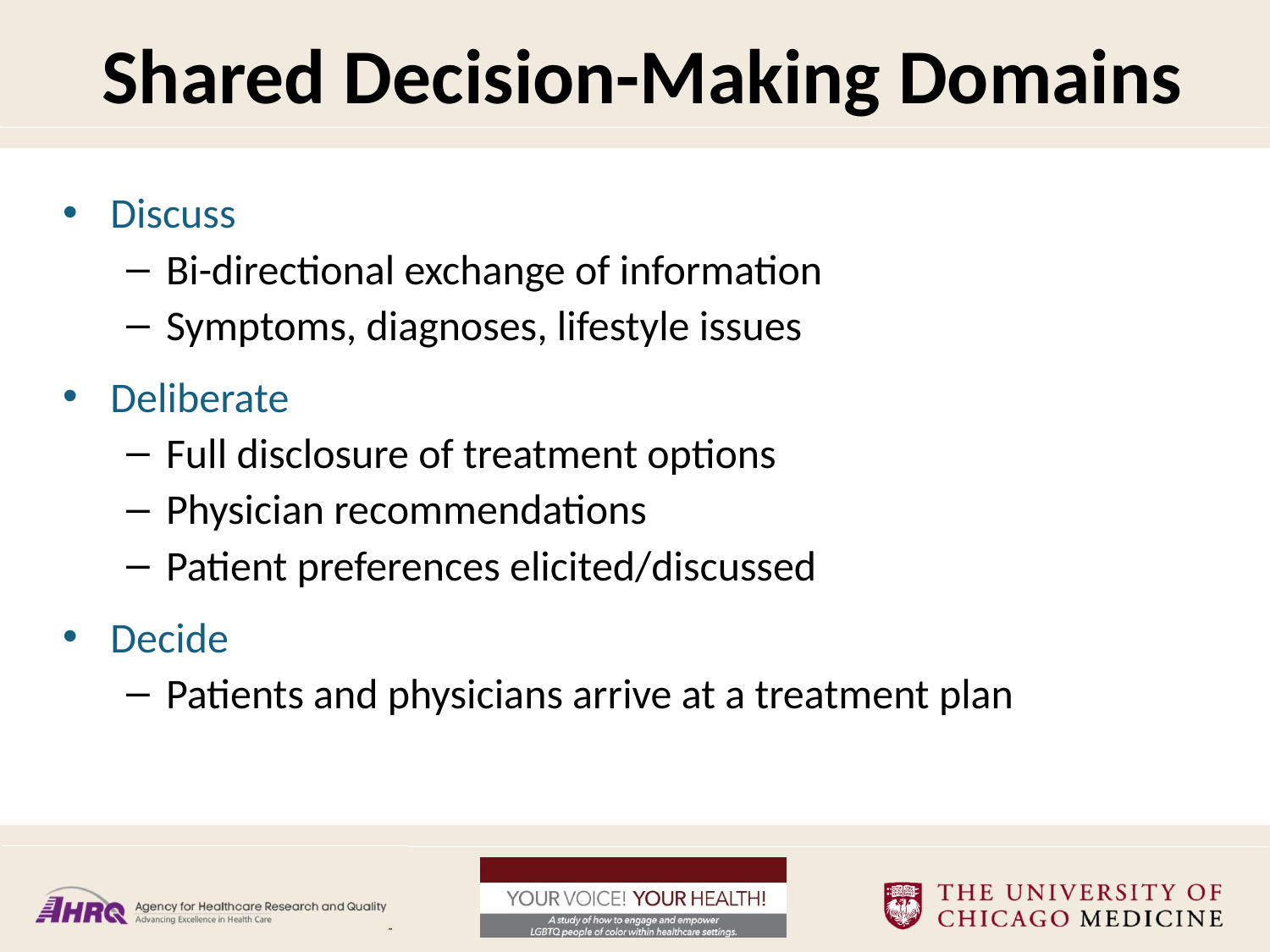

Shared Decision-Making Domains
Discuss
Bi-directional exchange of information
Symptoms, diagnoses, lifestyle issues
Deliberate
Full disclosure of treatment options
Physician recommendations
Patient preferences elicited/discussed
Decide
Patients and physicians arrive at a treatment plan

## Slide 24
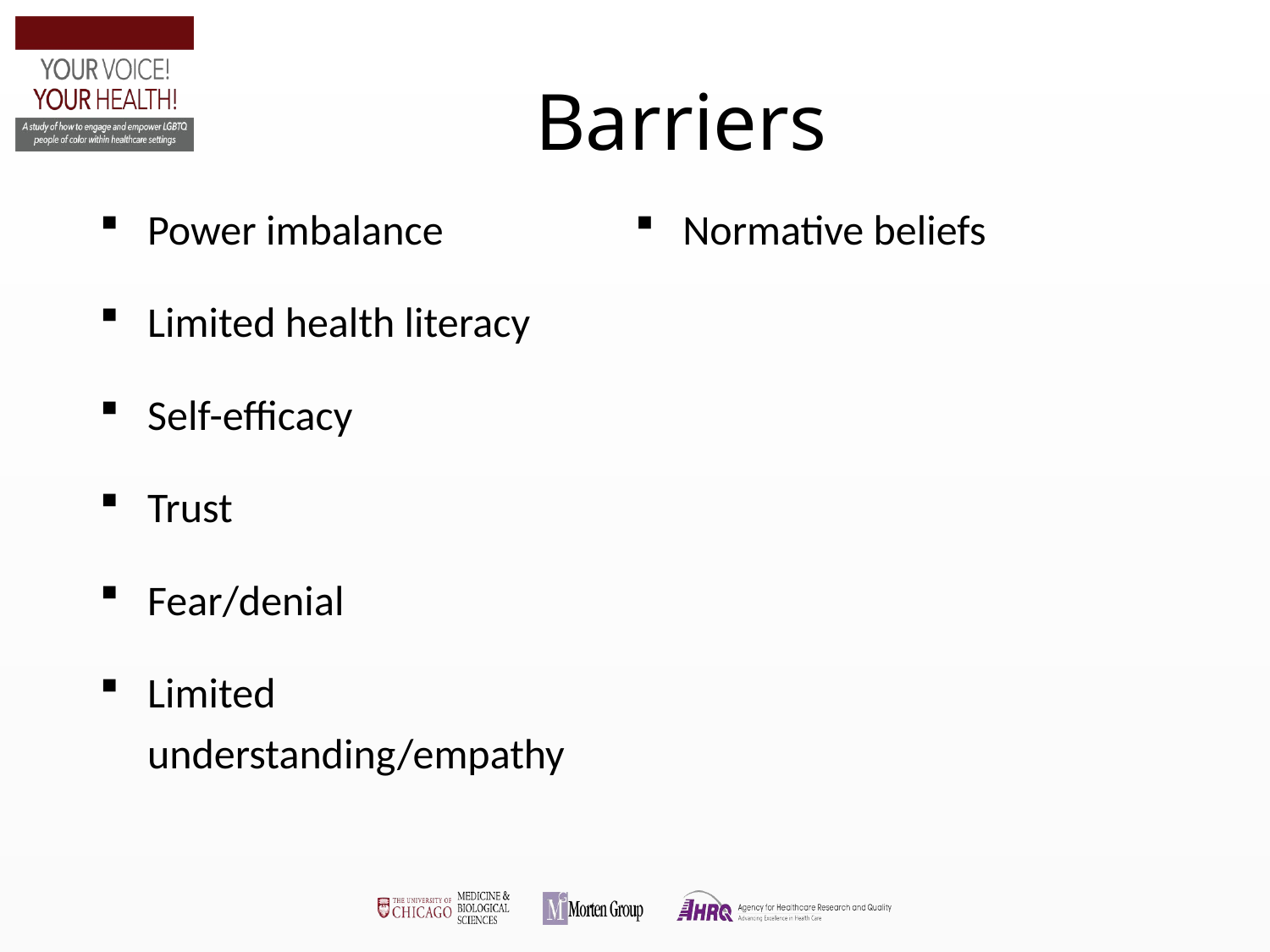

Barriers
Power imbalance
Limited health literacy
Self-efficacy
Trust
Fear/denial
Limited understanding/empathy
Normative beliefs

## Slide 25
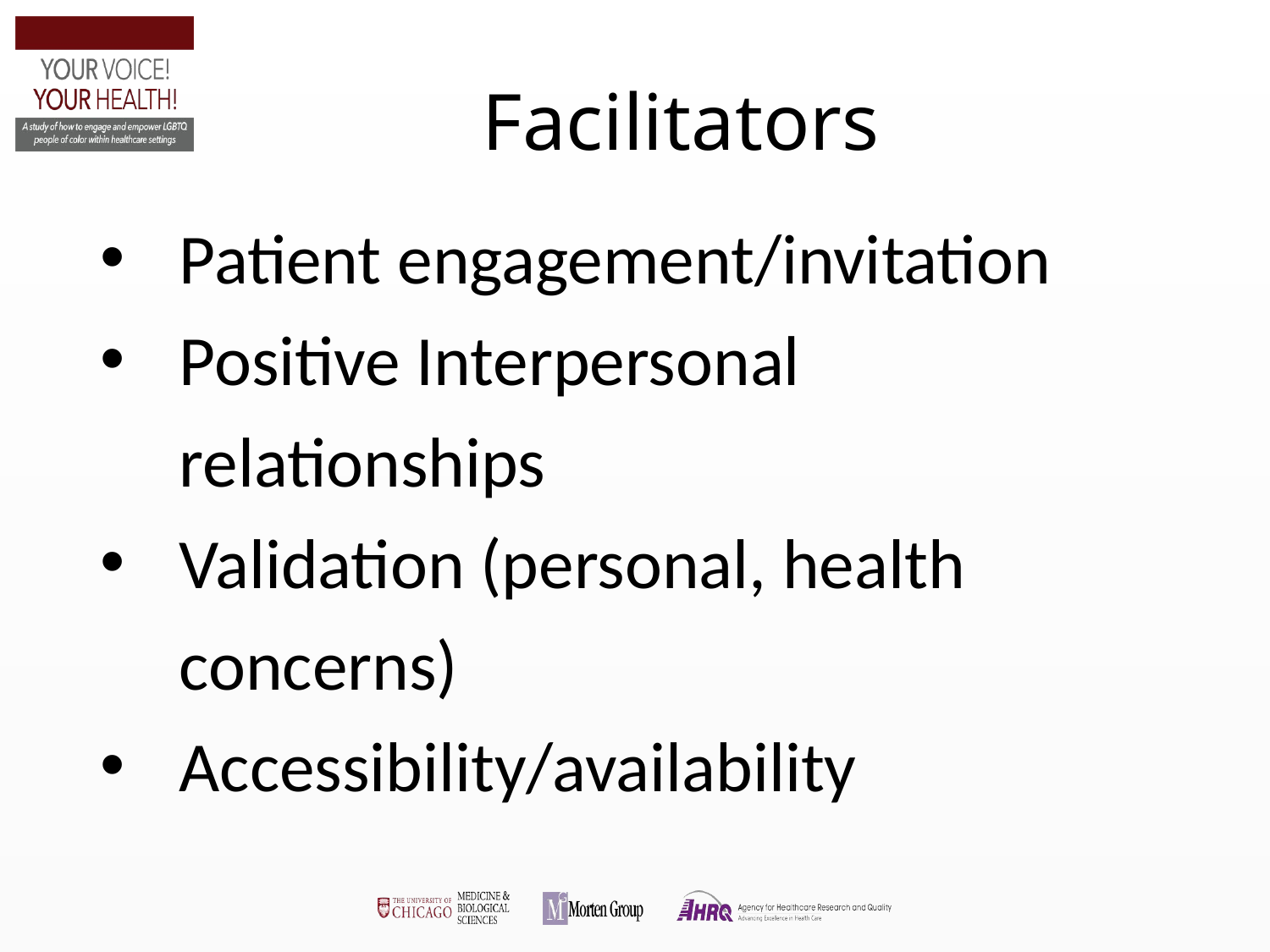

Facilitators
Patient engagement/invitation
Positive Interpersonal relationships
Validation (personal, health concerns)
Accessibility/availability

## Slide 26
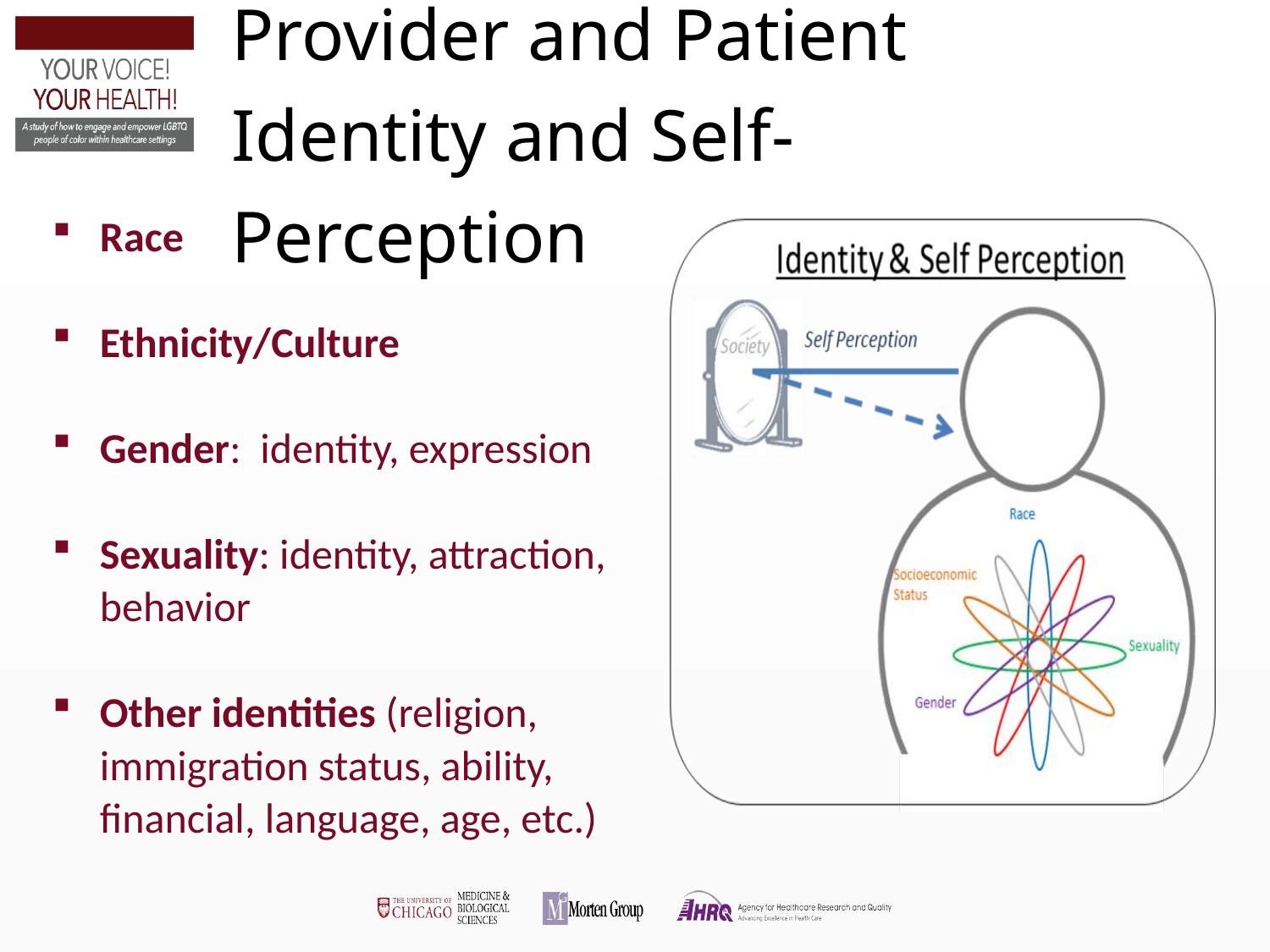

Provider and Patient
Identity and Self-Perception
Race
Ethnicity/Culture
Gender: identity, expression
Sexuality: identity, attraction, behavior
Other identities (religion, immigration status, ability, financial, language, age, etc.)

## Slide 27
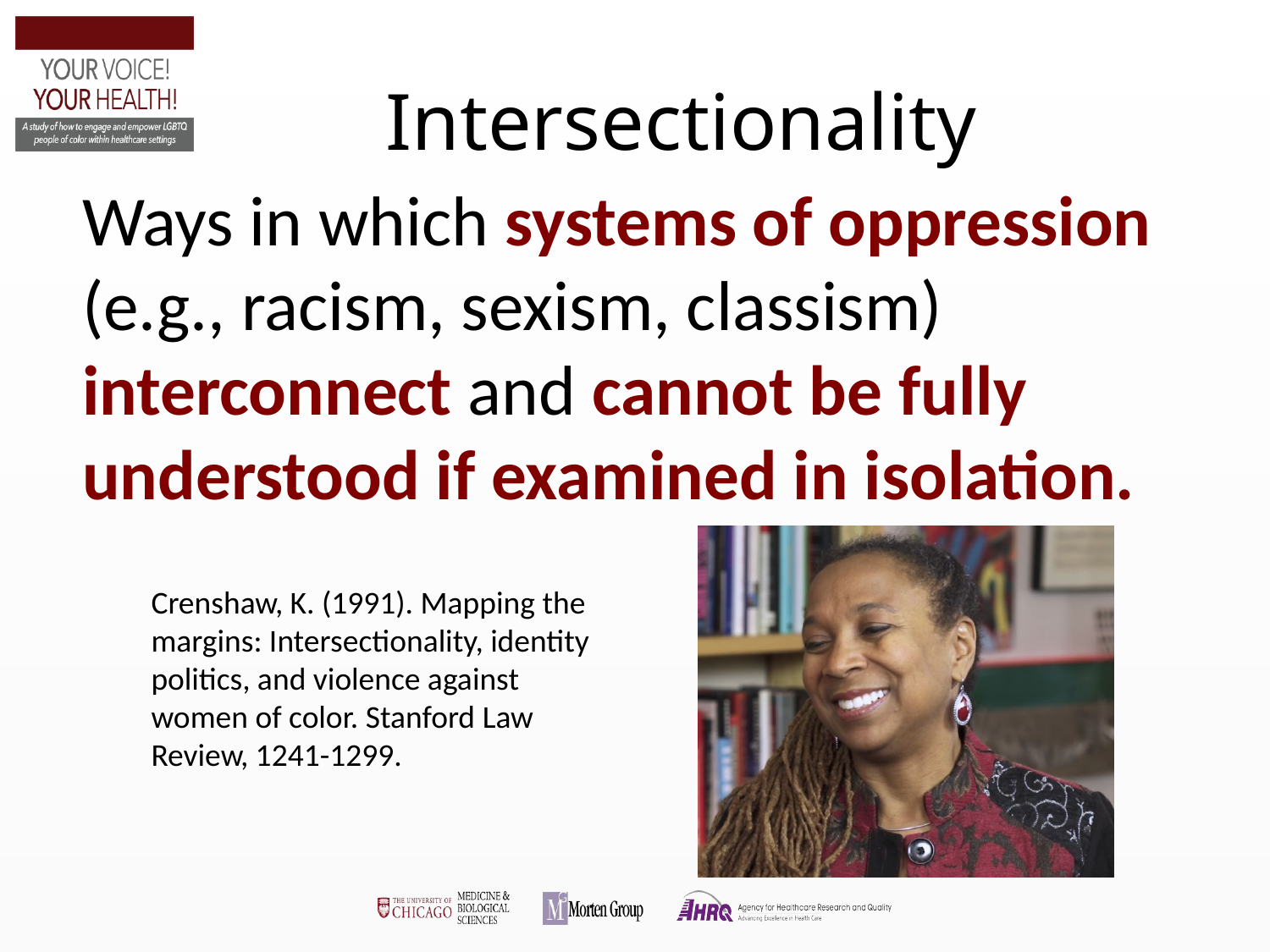

Intersectionality
Ways in which systems of oppression (e.g., racism, sexism, classism) interconnect and cannot be fully understood if examined in isolation.
Crenshaw, K. (1991). Mapping the margins: Intersectionality, identity politics, and violence against women of color. Stanford Law Review, 1241-1299.

## Slide 28
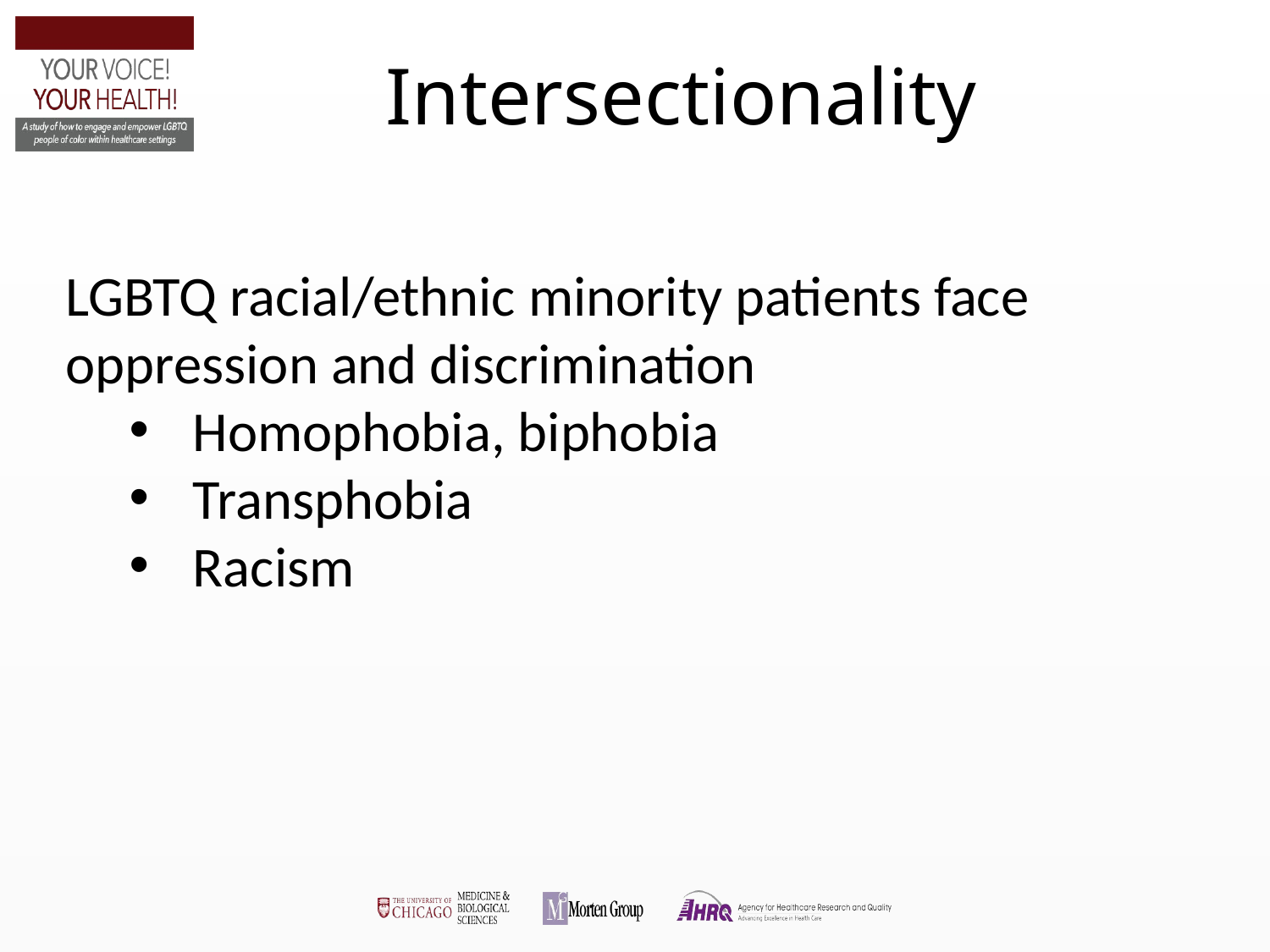

Intersectionality
LGBTQ racial/ethnic minority patients face oppression and discrimination
Homophobia, biphobia
Transphobia
Racism

## Slide 29
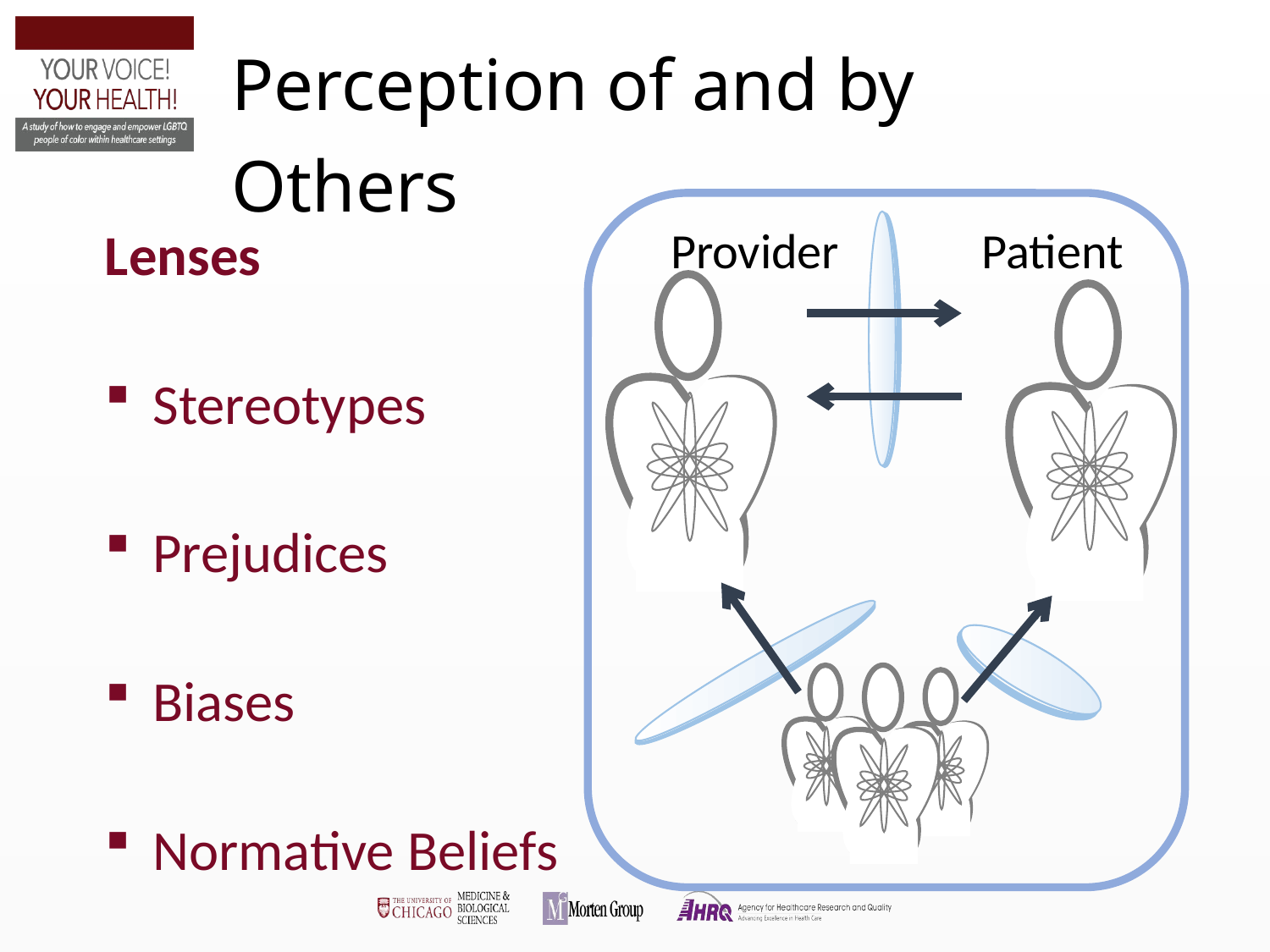

Perception of and by Others
Patient
Provider
0
0
0
0
0
0
0
0
0
0
Lenses
Stereotypes
Prejudices
Biases
Normative Beliefs

## Slide 30
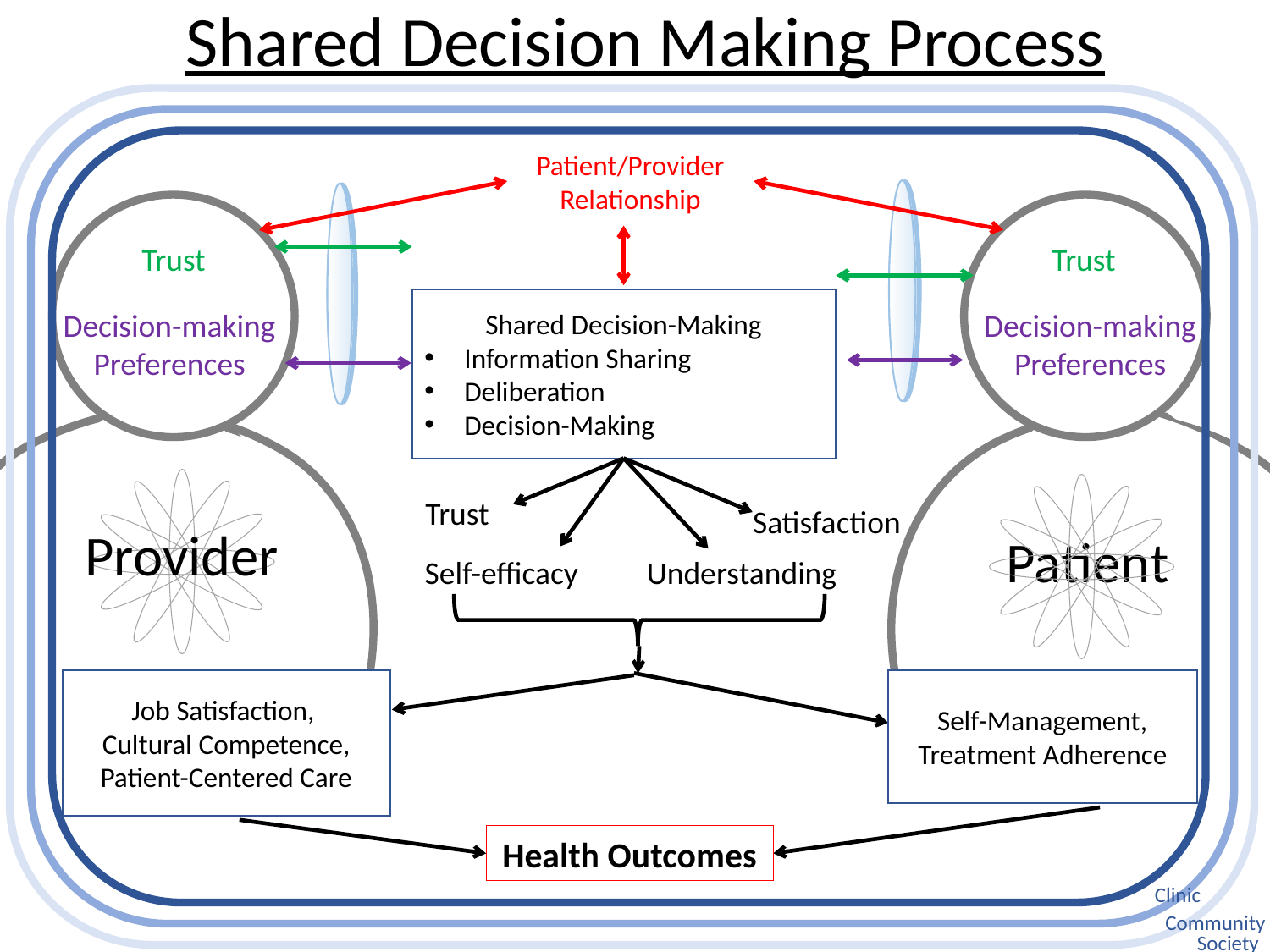

Shared Decision Making Process
Patient/Provider Relationship
0
0
0
0
Trust
Trust
Shared Decision-Making
Information Sharing
Deliberation
Decision-Making
Decision-making Preferences
Decision-making Preferences
Trust
Satisfaction
Provider
Patient
Self-efficacy
Understanding
Job Satisfaction,
Cultural Competence, Patient-Centered Care
Self-Management, Treatment Adherence
Health Outcomes
Clinic
Community
Society

## Slide 31
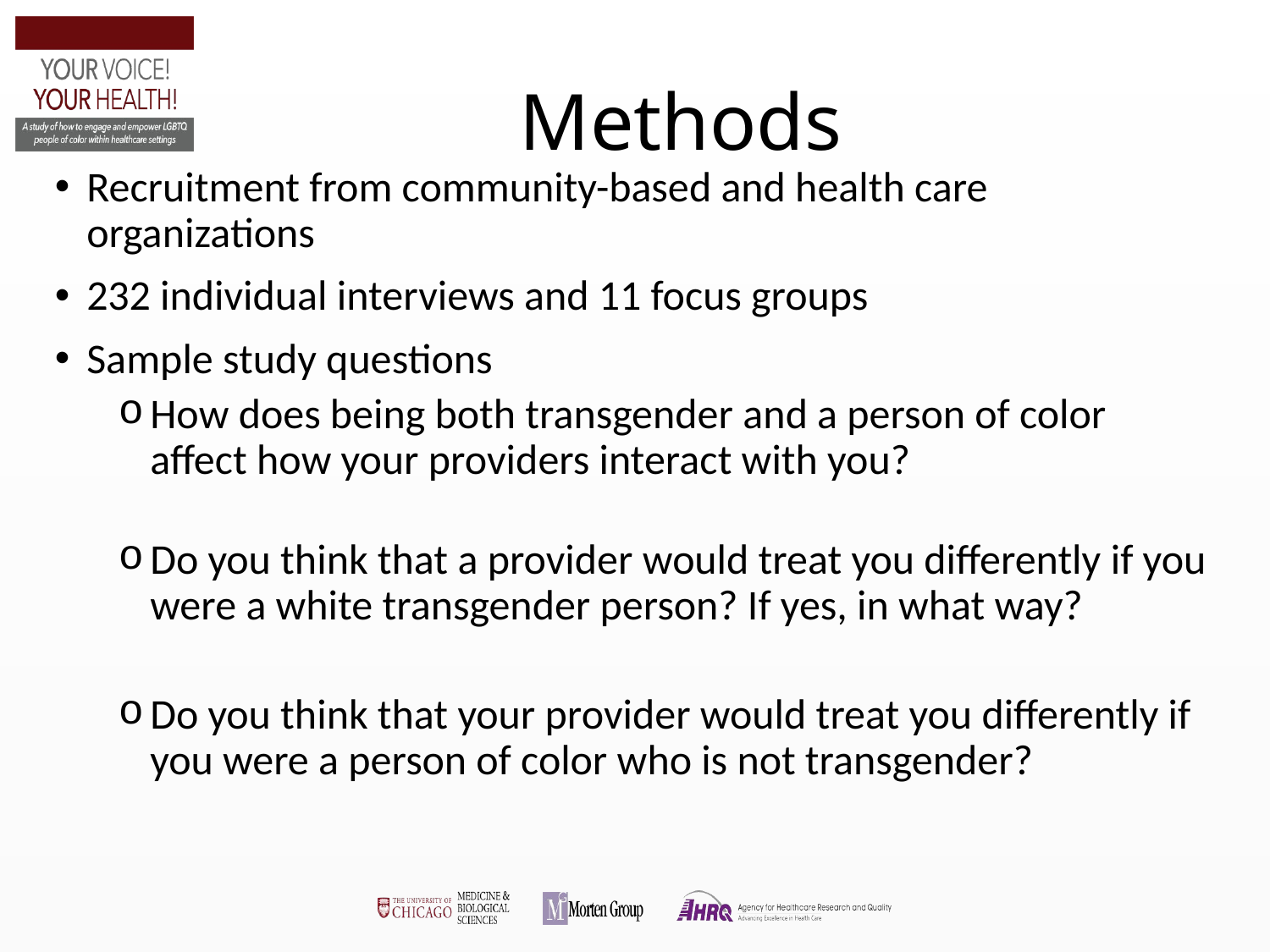

Methods
Recruitment from community-based and health care organizations
232 individual interviews and 11 focus groups
Sample study questions
How does being both transgender and a person of color affect how your providers interact with you?
Do you think that a provider would treat you differently if you were a white transgender person? If yes, in what way?
Do you think that your provider would treat you differently if you were a person of color who is not transgender?

## Slide 32
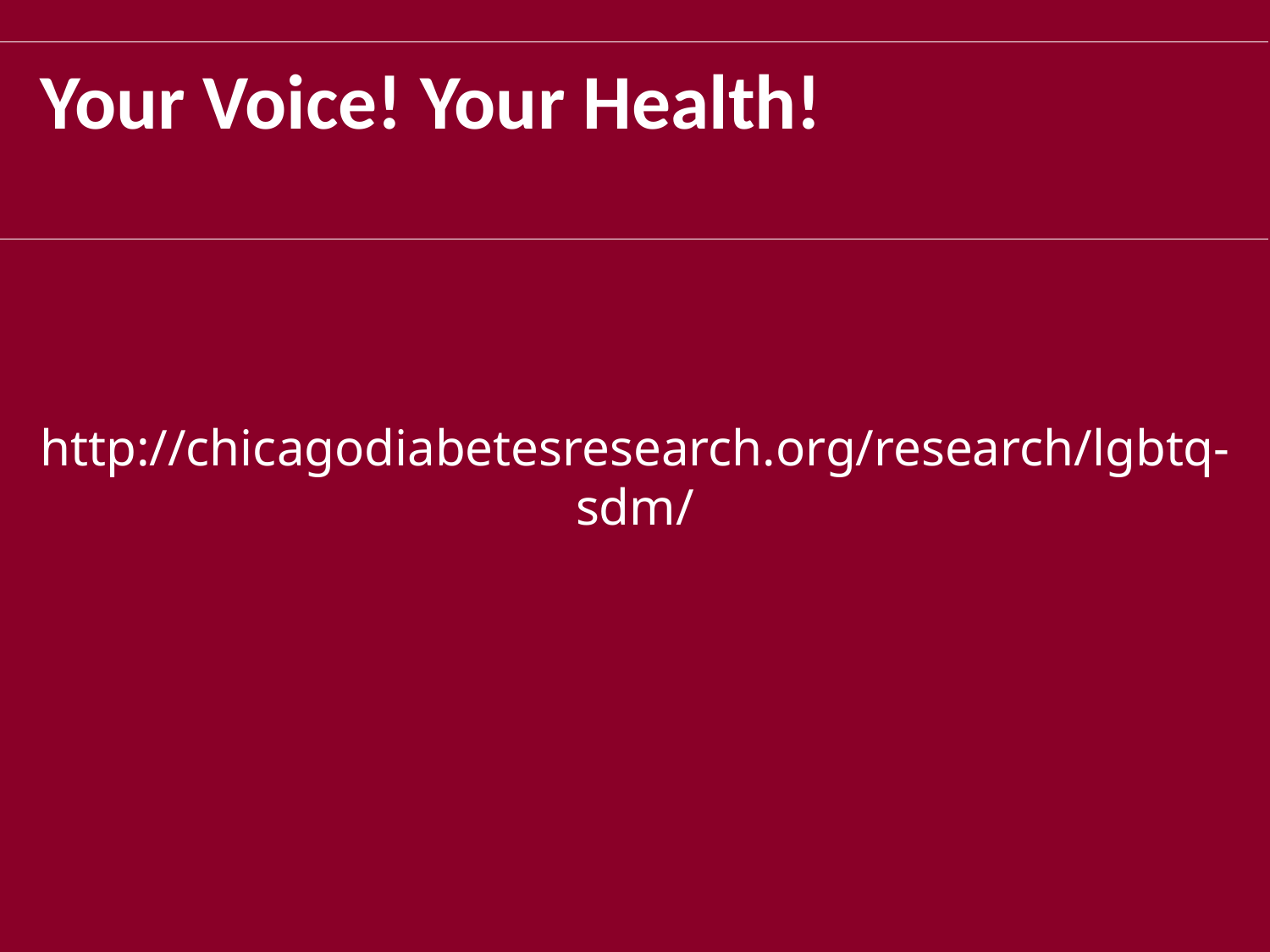

http://chicagodiabetesresearch.org/research/lgbtq-sdm/
Your Voice! Your Health!

## Slide 33
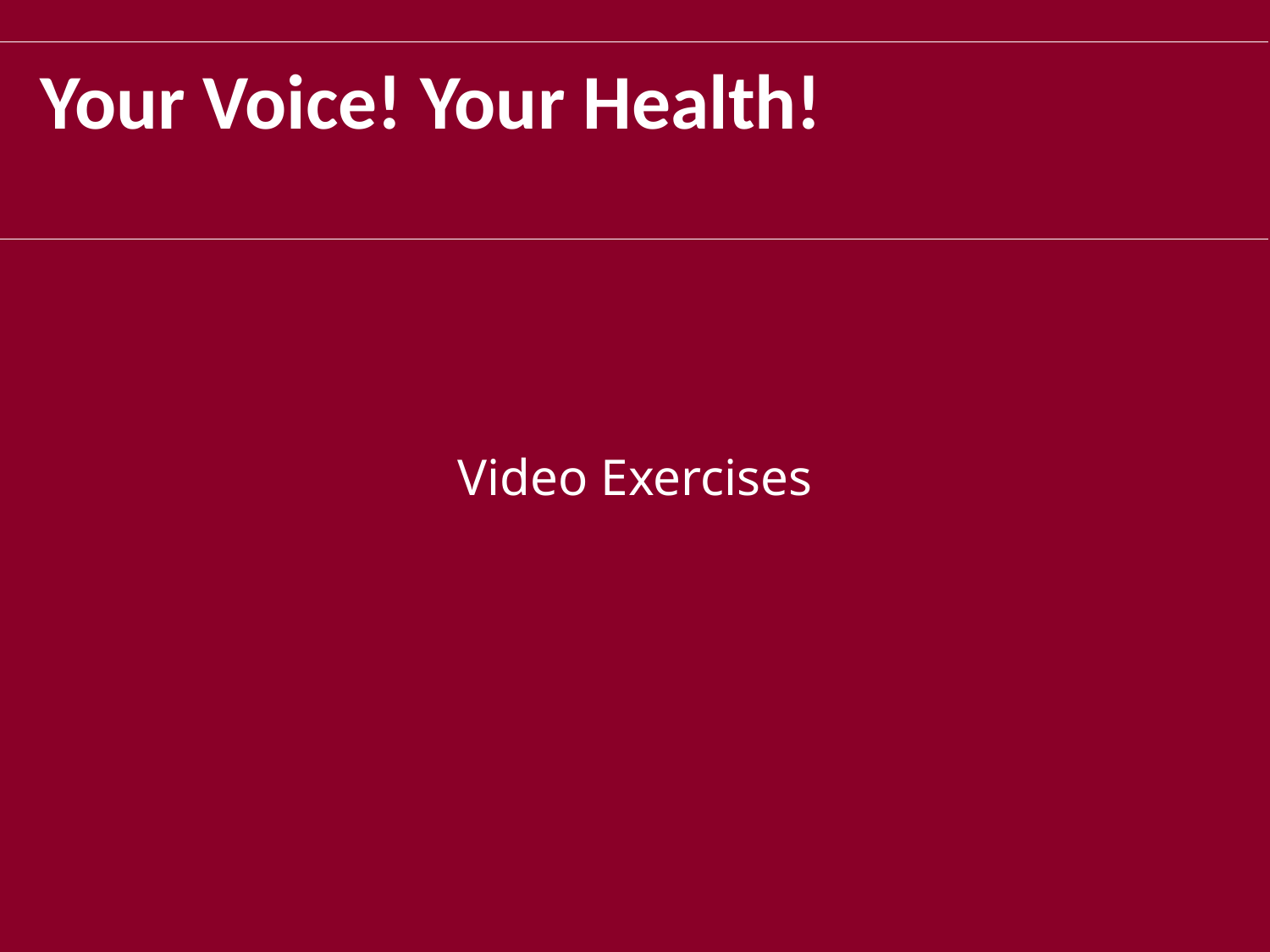

Video Exercises
Your Voice! Your Health!

## Slide 34
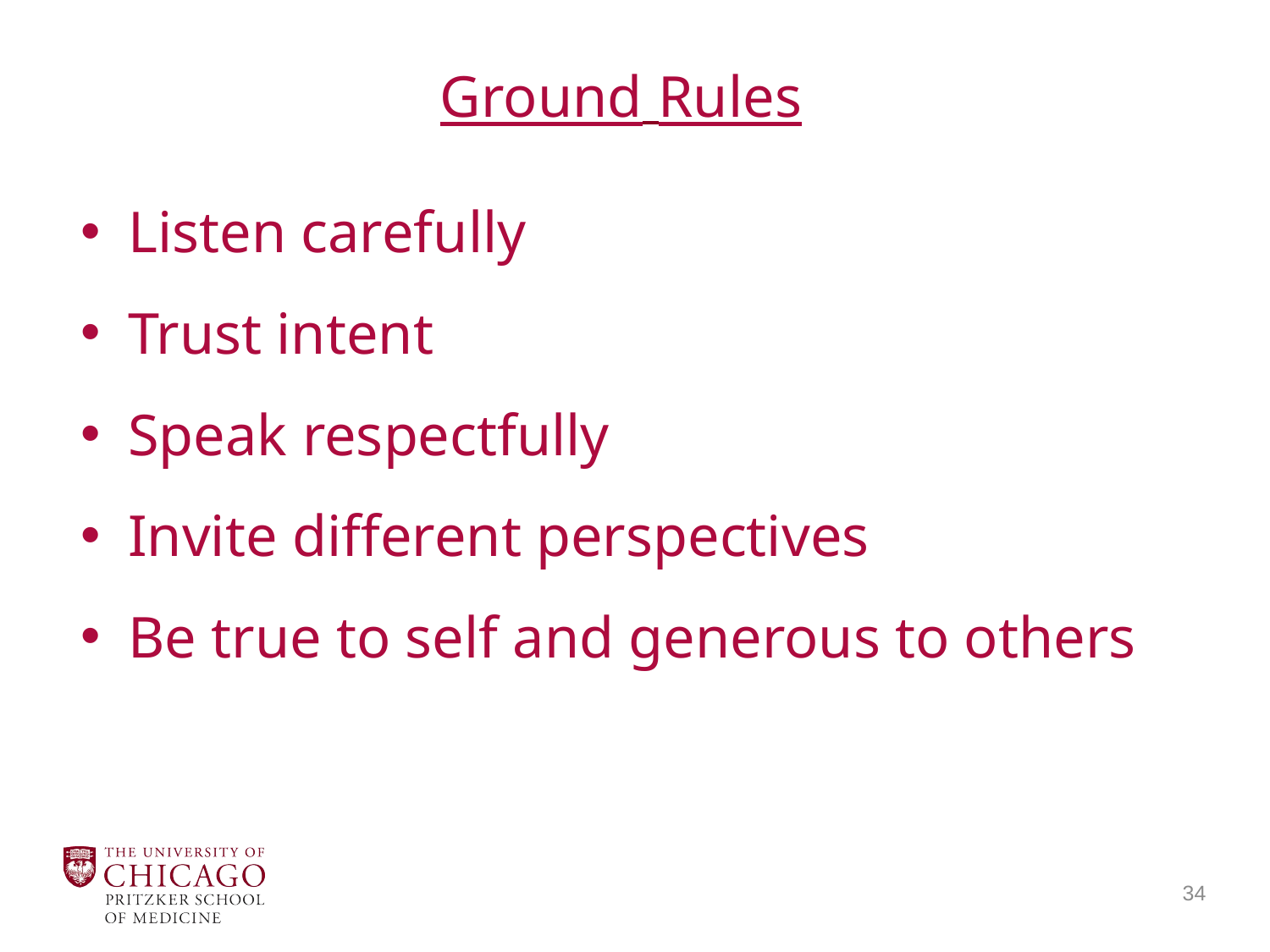

Ground Rules
Listen carefully
Trust intent
Speak respectfully
Invite different perspectives
Be true to self and generous to others
34
