# Supplementary material for: Teaching Intersectionality of Sexual Orientation, Gender Identity, and Race/Ethnicity in a Health Disparities Course
Source: MedEdPORTAL. 2020 Jul 31;16:10970. doi: 10.15766/mep_2374-8265.10970 (PMC7394350; doi:10.15766/mep_2374-8265.10970)
Supplement: Supplementary file 1 — Aurora Video.mp4Don Video.mp4Reyna Video.mp4Vita Video.mp4Sam Video.mp4Intersectionality Lecture.pptxSuggested Discussion Questions.docxPre- and Postsurveys.docx [file mep_2374-8265.10970-s001.zip › G. Suggested Discussion Questions.docx]

Suggested discussion questions for patient videos:

**For All Videos**

What did you find particularly remarkable about X’s story?

Did anything surprise you about this video?

How was X’s racial/ethnic identity important? How was X’s LGBTQ identity important? How did X describe their intersecting identities with reference to specific lived experiences or healthcare encounters?

Under what circumstances were some identities more important to X than others?

Are there identities separate from race/ethnicity and LGBTQ identity that were important in X’s story?

Reflect on your own biases surrounding LGBTQ people of color. Have the videos made you more aware of any of these biases?

**Aurora** - Latina lesbian gender non-conforming individual with a history of obesity/body image issues

How did being Latina affect Aurora’s experience in the LGBTQ community?

How did Aurora’s sexual and gender identities affect her relationship with the Latinx community?

What makes it difficult for Aurora’s mother to accept her?

Aurora says she is very feminine even though she looks the way she does. What does she mean by this? (Note to facilitator: She seems to be making a distinction here between gender identity and gender expression.)

What was important for Aurora in feeling connected to her identities/communities?

How does disclosing SGM identity affect the health of different individuals differently?

**Don** - Older African American gay man

In what ways has Don’s identities affected his life?”

Don says he is always viewed as a “black gay man,” never just a “gay man.”

What did he mean by that?

What are some resources and privileges that white gay men have that black gay men do not?

What sort of experiences do LGBTQ older adults like Don have that are different than today’s LGBTQ youth?

What do you think it may feel like for patients of color to leave their community?

For health care?

For social activities?

**Vita** - African American transfemme individual with chronic health conditions

How does this clip demonstrate minority stress?

(Note to facilitator: Minority stress is defined as “chronically high levels of stress faced by members of stigmatized minority groups” which can be amplified if you belong to multiple minority groups, like Vita does.)

How did you feel when Vita mentioned that doctors seem to have a “sense of entitlement?”

Vita mentioned making information more accessible to patients, rather than using jargon. How do you think this relates back to identities, if at all?

**Reyna** - Latina transsexual woman

What are some clinic-level policies or best practices that you would advocate for that could improve future experiences for Reyna and other trans women of color (TWOC)? (See DeMeester RH, Lopez FY, Moore JE, Cook SC, Chin MH. A model of organizational context and shared decision making: application to LGBT racial and ethnic minority patients. J Gen Intern Med 2016; 31:651-62. doi: 10.1007/s11606-016-3608-3.)

How would you address stereotypes of TWOC as human immunodeficiency virus (HIV)-positive and sexually transmitted infection (STI)-positive?

**Sam** (skipped in our session due to time) - Asian American transmasculine individual who was a survivor of intimate partner violence

Sam mentions a spectrum within transmasculine. Who has heard of the term transmasculine? How would you define transmasculine?

What are some barriers for Sam in discussing intimate partner violence (IPV)?

When Sam talks about “Stereotypes about who can and cannot perpetrate harm, what does he mean by this? What kind of relationships do we usually think of when we think of IPV?
