# Supplementary material for: Teaching Intersectionality of Sexual Orientation, Gender Identity, and Race/Ethnicity in a Health Disparities Course
Source: MedEdPORTAL. 2020 Jul 31;16:10970. doi: 10.15766/mep_2374-8265.10970 (PMC7394350; doi:10.15766/mep_2374-8265.10970)
Supplement: Supplementary file 1 — Aurora Video.mp4Don Video.mp4Reyna Video.mp4Vita Video.mp4Sam Video.mp4Intersectionality Lecture.pptxSuggested Discussion Questions.docxPre- and Postsurveys.docx [file mep_2374-8265.10970-s001.zip › H. Pre- and Postsurveys.docx]

**Pre-Session Survey**

| **How confident do you feel in your ability to…** | **Not at all confident**  **0** | **Not very confident**  **1** | **Neutral**  **2** | **Somewhat confident**  **3** | **Completely confident**  **4** |
| --- | --- | --- | --- | --- | --- |
| Define the terms: sex, gender, sexual orientation, gender identity, and gender expression. | 0 | 1 | 2 | 3 | 4 |
| Define the term: intersectionality. | 0 | 1 | 2 | 3 | 4 |
| Define the term: minority stress. | 0 | 1 | 2 | 3 | 4 |
| Identify barriers to care for LGBTQ patients. | 0 | 1 | 2 | 3 | 4 |
| Identify ways for providers to help LGBTQ patients overcome barriers to care. | 0 | 1 | 2 | 3 | 4 |
| Ask LGBTQ patients about their identities. | 0 | 1 | 2 | 3 | 4 |
| Help connect an LGBTQ patient to the resources they may need. | 0 | 1 | 2 | 3 | 4 |
| What does intersectionality mean to you? | | | | | |

**Post-Session Survey**

| **Compared to prior to this module, how confident do you feel in your ability to…** | **Not at all confident**  **0** | **Not very confident**  **1** | **Neutral**  **2** | **Somewhat confident**  **3** | **Completely confident**  **4** |
| --- | --- | --- | --- | --- | --- |
| Define the terms: sex, gender, sexual orientation, gender identity, and gender expression. | 0 | 1 | 2 | 3 | 4 |
| Define the term: intersectionality. | 0 | 1 | 2 | 3 | 4 |
| Define the term: minority stress. | 0 | 1 | 2 | 3 | 4 |
| Identify barriers to care for LGBTQ patients. | 0 | 1 | 2 | 3 | 4 |
| Identify ways for providers to help LGBTQ patients overcome barriers to care. | 0 | 1 | 2 | 3 | 4 |
| Ask LGBTQ patients about their identities. | 0 | 1 | 2 | 3 | 4 |
| Help connect an LGBTQ patient to the resources they may need. | 0 | 1 | 2 | 3 | 4 |

| **Please rate the following:** | Poor  0 | Fair  1 | Good  2 | Very Good  3 | Excellent  4 |
| --- | --- | --- | --- | --- | --- |
| Lecture | 0 | 1 | 2 | 3 | 4 |
| Videos with discussion | 0 | 1 | 2 | 3 | 4 |
| Overall session | 0 | 1 | 2 | 3 | 4 |
| What does intersectionality mean to you? | | | | | |
| Please comment on the strengths of the session.  Please comment on any ways in which the session could be improved. | | | | | |

| **To what extent do you agree with the following?** | **Strongly disagree**  **0** | **Somewhat**  **disagree**  **1** | **Neutral**  **2** | | **Somewhat agree**  **3** | **Strongly**  **disagree**  **4** |
| --- | --- | --- | --- | --- | --- | --- |
| The workshop informed my future communication with patients. | 0 | 1 | 2 | | 3 | 4 |
| The workshop motivated me to want to take action in addressing LGBTQ health disparities. | 0 | 1 | 2 | | 3 | 4 |
| I want to tell others about what was presented. | 0 | 1 | 2 | 3 | | 4 |
